# Combining individual and close‐kin mark–recapture to design an effective wildlife population survey

**DOI:** 10.1002/ecy.70377

**Published:** 2026-06-22

**Authors:** Eiren K. Jacobson, Mark V. Bravington, Rebecca L. Taylor, Irina S. Trukhanova, David L. Miller, William S. Beatty

**Affiliations:** ^1^ Centre for Research into Ecological & Environmental Modelling and School of Mathematics & Statistics, University of St Andrews St Andrews UK; ^2^ Estimark Research Hobart Australia; ^3^ US Geological Survey Alaska Science Center Anchorage Alaska USA; ^4^ North Pacific Wildlife Consulting, LLC Seattle Washington USA; ^5^ US Fish and Wildlife Service, Marine Mammals Management Anchorage Alaska USA; ^6^ Independent Researcher Dundee UK; ^7^ US Geological Survey Upper Midwest Environmental Sciences Center La Crosse Wisconsin USA

**Keywords:** close‐kin mark–recapture, individual genetic mark–recapture, survey design, walrus

## Abstract

Close‐kin mark–recapture (CKMR) is a promising approach for assessing population size of species that have been difficult to survey using more traditional methods. Here, we combine individual and close‐kin mark–recapture in a single modeling framework (ICKMR) and provide an example of study design using this approach for Pacific walrus (*Odobenus rosmarus divergens*). We develop the ICKMR model and test it using simulated datasets, then use properties of the pseudo‐likelihood to investigate the expected precision in estimates of abundance with different proposed survey designs. Our motivating example, the Pacific walrus, is an ice‐associated marine mammal found in the Bering and Chukchi seas, where it is an important resource for Indigenous peoples. Pacific walrus abundance declined in the late 20th century, and it is currently a species of conservation concern due to potential impacts of climate change, particularly the loss of sea ice. To reduce uncertainty in population size estimates, researchers undertook a genetic mark–recapture sampling campaign from 2013 to 2017 and collected tissue samples from over 8000 individuals. Another campaign of a similar scale is ongoing (2023–2028). While sample collection was designed for individual mark–recapture, advances in CKMR methods and associated molecular techniques mean that these samples could also be suitable for CKMR. The advantages of CKMR over mark–recapture include an increased effective sample size (because each individual tags itself and its parents, siblings, and offspring) and additional insights into demographic quantities of interest. To make best use of genetic samples, we combine individual mark–recapture (IMR) with CKMR (ICKMR) and investigate whether different sampling strategies can increase precision in estimates of abundance. Our modeling approach includes special considerations for walrus life history, including a multi‐year inter‐birth interval. We found that expected coefficients of variation (CVs) of the ICKMR estimates of abundance, adult female survival, juvenile female survival, and proportion of breeding females are lower than those expected from IMR alone, and with ICKMR, fewer years of sampling can be conducted to obtain sufficient precision in estimates of abundance. This work demonstrates the utility of ICKMR and could be applicable across a variety of taxa.

## INTRODUCTION

Estimation of abundance and other demographic parameters such as survival are a key part of wildlife management and conservation. Traditional mark–recapture analysis (Williams et al., 2002) can deliver estimates with low bias and uncertainty, provided enough individual animals (1) are identifiable by natural, artificial, or genetic “marks” and (2) can be recaptured over time. If genotypes are used as marks, as in genetic individual mark–recapture (IMR; Palsbøll et al., 1997), then kinship patterns amongst samples (e.g., parents, siblings) contain additional demographic information (Skaug, 2001). Close‐kin mark–recapture (CKMR; refer to Bravington et al., 2016) is a framework for using pairwise kinships, as inferred from genotypes, to estimate abundance and demographic parameters. CKMR provides additional flexibility compared to conventional mark–recapture because it is not essential to recapture individuals, so lethal (e.g., from sampling, harvest, or natural mortality) and/or nonlethal samples can be used. As of 2025, most CKMR projects have focused on commercial fish (e.g., Davies et al., 2020) or sharks (e.g., Hillary et al., 2018), but a few have been conducted on mammals (e.g., Conn et al., 2020; Lloyd‐Jones et al., 2023; Taras et al., 2024).

The principle behind CKMR is that every individual has one mother and one father, so each captured individual “tags” itself and its parents. For a given sample size, a large population is expected to have fewer “recaptures” of closely related individuals compared to a small population. In practice, CKMR data are derived from pairwise comparisons among samples while considering covariates such as age, size, and sex. Each pair of samples is tested for a series of kinship relationships such as parent–offspring, half‐sibling, or self (the alternative being “unrelated,” i.e., none of the above). The CKMR model has two components: (1) a population dynamics model driven by the demographic parameters; and (2) formulae for expected frequencies of different kinship types in pairwise comparisons that are conditional on sample covariates and demographic parameters. By combining the kinship data with the population dynamics model, parameters can be estimated using maximum likelihood or Bayesian methods.

The success of CKMR depends on whether the data collected contain enough close‐kin pairs to yield acceptable precision in parameter estimates. The chance of success is greatly increased by a study design exercise that evaluates the effects of sample age, size, and sex composition of sampled animals, precision of covariate measurements, and study duration, while taking into account the species' life history, ecology, and physiology (Merriell et al., 2024; Petersma et al., 2024; Sévêque et al., 2024; Swenson et al., 2024; Waples & Feutry, 2022). The pairwise comparison framework leads to analytical results for the expected number of kin pairs and the parameter estimation variance, given the number of samples and associated covariates, so that simulation is not essential for study design. Nevertheless, simulation can be used to check kinship probability formulae, robustness to model simplifications, and design setup.

In this study, we perform and verify CKMR design calculations for the Pacific walrus (*Odobenus rosmarus divergens*; hereafter, walrus) to demonstrate the design process and the utility of CKMR. Our goal was to understand how different possible demographic scenarios and design choices would impact precision of estimates of adult female abundance, adult female survival, and juvenile survival. In addition, in most CKMR applications to date, self‐recaptures were unlikely or impossible (e.g., when sampling is lethal). Lloyd‐Jones et al. (2023) included individual mark–recapture results in a CKMR study but did not integrate both datasets into a single model. Bravington et al. (2014) extended CKMR to include self‐recaptures as an additional kinship type (individual‐ and close‐kin mark–recapture; ICKMR), whereby pairwise genetic comparisons can show that two samples are from the same animal. They found that the expected coefficient of variation (CV) in estimates of abundance decreased from approximately 30% with conventional mark–recapture alone to approximately 20% with ICKMR. We refer to models developed within a CKMR framework that only use self kin pairs as IMR. Here, we explore design scenarios using IMR alone versus a combined ICKMR approach, and we find that the latter can be used to reduce the amount of survey effort required for adequate monitoring.

### Pacific walrus biology and background

The Pacific walrus is a gregarious, ice‐associated pinniped inhabiting continental shelf waters of the Bering and Chukchi Seas. During winter (when sea ice forms south of the Bering Strait), almost all walruses occupy the Bering Sea (Fay, 1982). In summer (when sea ice is absent from the Bering Sea) almost all juvenile and adult female walruses, and some adult male walruses, migrate north to the Chukchi Sea. When walruses rest offshore on sea‐ice floes, their distribution is dynamic because it generally follows the marginal ice zone, which is a moving, changing habitat which contains a mix of ice floes and open water. Pacific walruses are considered a single, panmictic population (Beatty et al., 2020) and are managed as a single stock (US Fish and Wildlife Service, 2023). Adult female walruses move between US and Russian waters of the Chukchi Sea over the course of a single season (Jay et al., 2012; Udevitz et al., 2017). Female walruses breed in winter and give birth to a single calf approximately 14–15 months later (Fay, 1982; Robeck et al., 2022). Mothers and calves maintain a close physical relationship for the first year, and weaning generally occurs between the first and second birthday (Fay, 1982), though juveniles may travel with their mother until 3 years of age (Beatty et al., 2020). Female walruses may have their first calf at 6 years of age; male walruses do not reach sexual maturity until 15 years of age (Fay, 1985). Maximum walrus lifespan is approximately 40 years (Fay, 1982). Walruses can be aged from their teeth (Kryukova, 2014) and work is ongoing to develop an accurate epigenetic clock for walrus biopsy samples (i.e., estimated ages within 10% of true values; Robeck et al., 2023).

Abundance and demographic rate estimates are crucial for understanding population status and trends, as well as for co‐developing harvest management plans. Continued sea‐ice loss and a concomitant increase in the intensity and expansion of industrial and shipping activities in Pacific Arctic waters (Silber & Adams, 2019) are expected to drive a substantial population decline (Johnson et al., 2024, 2025; MacCracken et al., 2017; US Fish and Wildlife Service, 2011). Subsistence walrus harvests in Alaska and Chukotka exceed 4000 animals annually (US Fish and Wildlife Service, 2023), and Indigenous peoples and co‐management agencies need information on the status of the walrus population to manage these harvests sustainably. Furthermore, in the United States, the Marine Mammal Protection Act (MMPA; P.L. 92–522; 16 U.S.C. §§1361–1423 h) requires a determination of potential biological removal for walruses, which, in turn, requires a precise abundance estimate (Gilbert, 1999; Wade & DeMaster, 1999).

Scientists have attempted to ascertain walrus population size since at least 1880 (Fay et al., 1989), and until very recently, the most concerted effort was the 1975–2006 range‐wide aerial surveys conducted collaboratively by the USA and the USSR, and later, the Russian Federation. However, abundance estimates from these surveys were biased and imprecise. Aerial surveys were abandoned after the 2006 survey which yielded a 95% CI of 55,000–507,000 animals and a coefficient of variation (CV) of 0.93 for the population abundance estimate of 129,000 despite a rigorous design, innovative field methods, and sophisticated analyses. The imprecision in the estimate resulted from the walrus population being widely dispersed with unpredictable local clumping (Jay et al., 2012; Speckman et al., 2011). The first rigorous walrus survival rate estimates were obtained via Bayesian integrated population models (IPMs), which combined multiple data sources to estimate demographic rates and population trends over multiple decades (Taylor et al., 2018; Taylor & Udevitz, 2015). However, problems with the aerial survey data continued to preclude conclusions about population abundance in the IPMs (Taylor & Udevitz, 2015).

In 2013, the US Fish and Wildlife Service (FWS) began a genetic mark–recapture project to estimate walrus abundance and demographic rates (Beatty et al., 2020; Beatty et al., 2022). Genetic “marking” via skin biopsy samples (Palsbøll et al., 1997) is preferable to traditional marking techniques because walruses are extremely difficult to handle physically. In five summer research expeditions, biologists tried to biopsy a representative sample of walruses. In 2013, 2014, and 2016, biopsy samples were collected in US waters; in 2015 and 2017, biopsy samples were collected in both US and Russian waters (Beatty et al., 2022). Sampling focused on groups of adult females and juveniles because these classes are the demographically important population segments of this polygynous species (Beatty et al., 2022; Fay, 1982). Further details are given in Beatty et al. (2020) and Beatty et al. (2022). Data analysis from the 2013–2017 expeditions used a Cormack‐Jolly‐Seber multi‐event model to estimate survival rates, and a Horvitz‐Thompson‐like estimator to obtain population size. The total abundance estimate of 257,000 had a 95% credible interval (CrI) of 171,000–366,000 (CV = 0.19; Beatty et al., 2022). Although this was more precise than historical estimates from aerial surveys (e.g., Speckman et al., 2011), the study required extensive investment of human and financial resources. Thus, FWS and the US Geological Survey (USGS) initiated a second generation of expeditions in 2023 to estimate walrus abundance and vital rates with CKMR. Initially, expeditions were planned to occur each June from 2023 to 2027. A successful expedition to collect biopsy samples and other data was completed in 2023, but no samples were collected in 2024. Thus, we consider multiple study designs that extend from 2023–2028 rather than 2023–2027 (see *Scenarios*). Here we explore ICKMR as a way to substantially increase the information content compared to a model with self‐recaptures only without increasing sampling effort.

## METHODS

Our methods consist of two main components: ICKMR model development and simulation for model checking and design scenarios. To develop the ICKMR model, we selected the kinship categories that should be common in the samples as well as informative about recent abundance, built a walrus‐specific population dynamics model capable of handling those kinships, formulated pairwise kinship probabilities, defined the pseudo‐likelihood, and performed design calculations. We then developed an individual‐based simulation with walrus life history, used this simulation to check our ICKMR model, and generated simulated datasets from different demographic scenarios of interest with which we evaluated expected precision in parameter estimates under different possible survey designs.

### ICKMR model development

#### Kinship types

First, we considered which types of kinship may occur and are possible to detect in the population of interest given our knowledge of life history, resolution of genetic methods, and survey design. CKMR datasets often contain multiple genetically detectable kinships, all potentially providing some information about population dynamics. Potential kinships fall into three basic categories (though refer to *Discussion*): parent–offspring pairs (POPs); full‐sibling pairs (FSPs); and second‐order kin pairs (2KPs, comprising half‐sibling pairs (HSPs), grandparent–grandchild pairs (GGPs), and full‐thiatic pairs (FTPs), such as aunt–nephew). Not all those possible kinships would be informative about the most important aspects of walrus dynamics: abundance, trend, and mortality.

We ignored paternal HSPs and father–offspring pairs (FOPs) and did not model adult males at all because of irresolvable confounding between adult male abundance and persistent variability in breeding success (which is likely given lek‐based breeding; refer to Appendix S1: Section S1 for more details). We did not include within‐cohort HSPs or FSPs because walruses have a litter size of one and female walruses are unlikely to repeatedly mate with the same male. Similarly, we did not consider FTPs. GGPs are difficult to distinguish from HSPs, but we assume that they would be rare in our survey sample and could be excluded (refer to Appendix S1: Section S2). We are thus left with three kinships: mother–offspring pairs (MOPs), maternal half‐sibling pairs (XmHSPs), and self‐pairs (SPs) where an individual is captured at least twice and in different years. Note that male juvenile samples, which are common, are used as potential offspring or XmHSPs (i.e., they mark their mothers), but not as potential SPs or fathers. For model development and design, we assume that all kinship types are detected accurately.

#### Life history and population dynamics

##### Stage‐structured quasi‐equilibrium dynamics

In order to formulate kinship probability equations, CKMR requires a population dynamics model. In this case, we only needed to model females. We used a stage‐structured (juvenile/adult), rather than fully‐age‐structured approach because (1) most reproductive female adults are expected to have similar reproductive capacity and chance of survival and (2) stage‐structured models are simpler to implement for CKMR and require fewer parameters. Given these factors, and the broad goal of estimating adult female abundance, a stage‐structured model should be adequate for design purposes. We also opted not to include calves in the model because early life history survival (before animals are easily sampled) would require extra parameters that are difficult to estimate directly. In effect, calf survival and birth rate are combined into overall rate of change in abundance, which becomes a single parameter to be estimated.

The parameters to be estimated were adult female and juvenile female survival rates ($\phi_{A}$ and $\phi_{J}$), adult female abundance in some reference year ($N_{y,A}$), and population trend $\left(r\right).$ We used two stages: juveniles aged 1–5, and adults aged 6+ (the first age at which an accompanying calf is common). We assumed constant survival within each stage ($\phi_{A}$ and $\phi_{J}$). Because weaning occurs between 1 and 2 years of age (Fay, 1982) and most adult female mortality occurs during the subsistence harvest in the spring when 1‐year‐old walruses are nearly 2, we assumed that offspring survival from age 1 onwards was independent of its mother's survival. This is consistent with Taylor et al. (2018). While we assume that offspring survival is independent from age 1, we do not assume that sampling is independent until age 6 (refer to *Kinship probabilities*). Our model assumed that adult female abundance increased or decreased exponentially over the period covered by the population dynamics, which we set at 2000–2028. The model needs to cover all birth dates of samples that are used as potential offspring, XmHSPs, or SPs; choosing a lower limit of 2000 thus discards a few samples, but because there was more intense harvesting prior to 2000 and large changes in abundance (Taylor et al., 2018; Taylor & Udevitz, 2015) pre‐2000 age composition is difficult to model.

Adult female abundance in year y ($N_{y,A}$) is described by

(1) $$N_{y,A}=N_{y_{0},A}e^{r\left(y-y_{0}\right)},$$

where $e^{r}$ is the rate of population change and r=0 corresponds to stasis.

To incorporate self‐recaptures in the close‐kin model, we must assume stable age composition within stage. For that purpose, we assume that age composition over the modeled period is adequately described by the stable age or “quasi‐equilibrium” distribution consistent with survival $\phi_{A}$ and rate of change $e^{r}$. As shown in, for example, Keyfitz and Caswell (2005) Chapter 5, this is $N_{y,a}\;\propto \;N_{y,A}\phi_{A}^{a}e^{-ra}$.

##### The breeding cycle

Female walruses, like many other animals, exhibit “skip‐breeding,” taking gaps of one or more years between births. This intermittent breeding can cause bias in parameter estimates if not included in the CKMR model (Swenson et al., 2024; Waples & Feutry, 2022). Because XmHSPs will be important for walruses, we decided to include a sub‐model for skip‐breeding with parameters to be estimated from the CKMR data.

**FIGURE 1:**
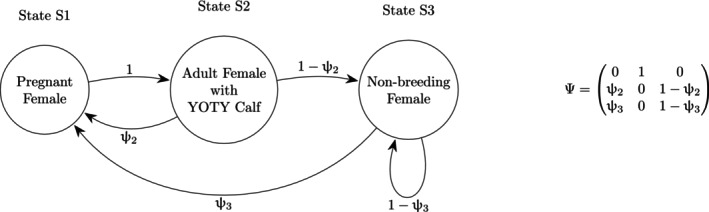
Directed cyclic graph showing the breeding cycle for walruses as represented in our Markov model. Nodes show the states (pregnant, with young‐of‐the‐year (YOTY), or quiescent) and edges give transition probabilities between those states. On average, female walruses reach sexual maturity (age of first ovulation) at age 4, so females enter the graph at the quiescent node. On the right is the transition matrix, Ψ, where cells indicate transition probabilities from row state to column state.

Walruses have a litter size of one, and due to a 14–15 month gestation, they cannot give birth in consecutive years (Fay, 1982; Katsumata et al., 2020; Robeck et al., 2022). They are also unlikely to give birth every second year (Fay et al., 1997; Robeck et al., 2023; Taylor et al., 2018; Taylor & Udevitz, 2015). We used a first‐order Markov model to describe the walrus breeding cycle (Figure 1). We assume three breeding states: (S1) pregnant; (S2) with young‐of‐the‐year (YOTY) calf; or (S3) mature and not in S1 or S2. From state S1 (pregnant), next year's state must be S2 (with YOTY calf). From state S2, a female may next year either return to state S1 (become pregnant again), with probability $\psi_{2}$, or move to state S3 (quiescent: neither pregnant nor with calf) with probability $1-\psi_{2}$. From state S3, she will either move to state S1 (become pregnant) with probability $\psi_{3}$, or remain in state S3 with probability $1-\psi_{3}$.

Females enter the breeding cycle at state S3 (i.e., reach sexual maturity) at age 4, and therefore sometimes become pregnant at age 5 and give birth at age 6 (Fay, 1982). Depending on the values of $\psi_{2}$ and $\psi_{3}$, this leads to fluctuations in effective fecundity (i.e., probability of being in state S2) over the first few years of adult life. Both $\psi_{2}$ and $\psi_{3}$ are estimated within the CKMR model, ultimately based on the observed distribution of birth gaps between XmHSPs. We do not use any data on whether females were with or without calf when sampled, and we cannot distinguish between fine‐scale aspects of the reproductive cycle, such as differences in fertilization/implantation rates versus pregnancy failures or neonatal deaths. Thus, because the transition from state S1 (pregnant) to state S2 (with YOTY calf) is set to 1, reproductive failures are subsumed by the fecundity rate.

We later use two quantities derived from the breeding cycle. First, we calculated the (average) proportion of adult females in S2 (with YOTY), ${\bar{\beta }}_{2}$. Let Ψ be the (3 × 3) transition matrix implied by the breeding cycle (Figure 1). Taking the eigen decomposition of Ψ, we extracted the second element of the eigenvector with the largest eigenvalue to obtain ${\bar{\beta }}_{2}$ (Caswell, 2001). Second, we defined fecundity as a function of age $B\left(a\right)$, where the numerator in Equation 2 gives the probability that any female of age a is in state S2. Thus,

(2) $$F\left(a\right)≜\frac{\mathbb{P}\left[B\left(a\right)=2\right]}{{\bar{\beta }}_{2}},$$

immature animals have fecundity 0, and an average adult has fecundity 1.

#### Kinship probabilities

Having formulated our population dynamics model, we now quantify the probability of relatedness between individuals. These probabilities will be used in the pairwise comparison terms summed in the pseudo‐log‐likelihood. The kinship probabilities are linked to the population dynamics model by shared parameters. For walruses, we considered three types of kinship: mother–offspring pair (MOP), cross‐cohort maternal half‐sibling pair (XmHSP), and self‐pair (SP), which represents individual recaptures in different years.

To establish demographic kinship probabilities between two sampled individuals, we apply the principle of expected relative reproductive output (ERRO; Bravington et al., 2016). For example, the probability that a given adult female is the parent of an independently sampled offspring is the ratio of that adult's expected fecundity to the total fecundity of all parents at the time the offspring was born. We denote the kinship for individuals i and j as $K_{\mathrm{ij}}$, which in our case may be MOP, XmHSP, SP, or unrelated pair (UP). Sampling was assumed to occur on an annual basis, that is, a maximum of one sample per individual per year would be used and any within‐year individual recaptures discarded. In the case of MOPs and XmHSPs, if recaptures exist in different years, we ensure that only one sample (the last) from each individual is used (so “sample” and “individual” are interchangeable terms). We can then be certain that the individual was alive until the time of the last sample, and so, conditional on age, could have produced offspring at least up until that time. In addition, using only the last sample improves precision in estimates of survival because the individual definitely survived to age at last sample. For SPs, we consider the first and last sample from each individual (in which case, “sample” and “individual” have different meanings) to maximize the interval over which we can be certain that the individual was alive.

We use the following notation: individual i, sampled at age $a_{i}$ in year $y_{i}$ with birth year $b_{i}≜y_{i}-a_{i}$. As noted above, we only estimate adult female abundance. However, we use notation that could be adopted in models that estimate both juvenile and adult female abundance. In our notation, $N_{y,A}$ refers to adult female abundance in year *y*. We include the A subscript for clarity throughout the manuscript. In our derivation of juvenile female abundance (Appendix S1: Section S3), we simply change the second subscript to J for juvenile, $N_{y,J}$. Thus, we generalize this notation to $N_{y,d}$ where y denotes year and d denotes developmental stage (A = adult or J = juvenile). We define the binary variable L to indicate lethality of sampling ($L_{i}=1$ indicates lethal sampling for sample i). We use $I\left(\right)$ as an indicator function, returning 1 when the condition inside the brackets is true, else 0. Kinship probabilities are functions of demographic parameters such as $\phi_{A}$ and $N_{y_{0},A}$; we use θ as shorthand for this set of parameters, which become explicit in later iterations of the formulae.

We assumed that epigenetic age estimates will be available for all samples, based on an epigenetic aging approach (Polanowski et al., 2014; Robeck et al., 2023). Our model could be extended to incorporate errors in estimated age (with standard deviation assumed known, that is, after calibration of epigenetic age to known‐age samples), though the results here assume no errors; refer to the *Discussion* section.

##### Mother–offspring pairs (MOPs)

Consider a comparison between a potential mother *i* and a potential offspring *j*. We restrict our analysis to comparisons that satisfy the following:

1. i is female (though j need not be);
2. $a_{j}⩾1$ (no YOTY samples are used);
3. $y_{i}\neq y_{j}\vee a_{j}\geq a_{b}$ (do not compare potential offspring and mothers sampled in the same year unless offspring age is at least the age of first birth, $a_{b}$ = 6);
4. $b_{j}⩾2000$ (birth year of potential offspring must be greater than or equal to 2000, because population dynamics starts at year 2000).

Walruses may accompany their mothers until age 3 (Beatty et al., 2020). To ensure independence, we do not compare potential offspring that are juveniles (≤5 years old) to potential mothers sampled in the same year. However, we do compare potential offspring that are adults (≥6 years old) to potential mothers sampled in the same year. Furthermore, we compare all potential offspring to all potential mothers sampled in different years, regardless of age, because we assume that the sampling events are independent.

We can now distinguish two cases: $y_{i}<b_{j}$ (potential mother sampled before potential offspring birth) and $y_{i}⩾b_{j}$ (potential mother sampled after potential offspring birth).

For $y_{i}<b_{j}$, individual *i* still has to survive one or more years in order to be individual *j*'s mother (note that *i* may be immature when sampled, but mature by the time of *j*'s birth). In this case, i's sampling *must* be nonlethal ($L_{i}=0$). The MOP probability is

(3) $$\mathbb{P}\left[K_{\mathrm{ij}}=\mathrm{MOP}|a_{i},y_{i},b_{j},L_{i}=0,\theta \right]=\frac{R_{i}\left(b_{j}|y_{i},a_{i}\right)}{R^{+}\left(b_{j}\right)}$$

where $R_{i}\left(b_{j}|y_{i},a_{i}\right)$ is the expected reproductive output (ERO) of individual i in year $b_{j}$ given i is age $a_{i}$ in year $y_{i}$. For the denominator, $R^{+}\left(b_{j}\right)$ is the total reproductive output (TRO) of the whole population in year $b_{j}$. ERO and TRO are in units of “number of calves” (males and females) here (though generally their units are arbitrary but matching). TRO is the total number of adult females in the population when j is born, $N_{b_{j}A}$, multiplied by the proportion of females with calves (breeding state S2), ${\bar{\beta }}_{2}$: $R^{+}\;\left(b_{j}\right)={\bar{\beta }}_{2}N_{b_{j},A}$.

For the numerator, i's ERO has two components: first, she has to survive; second, she has to be calving (breeding state S2) in $b_{j}$:

(4) $$R_{i}\left(b_{j}|y_{i},a_{i}\right)=\Phi \left(b_{j}-y_{i},a_{i}\right)\mathbb{P}\left[B\left(a_{i}+b_{j}-y_{i}\right)=2\right],$$

where $\Phi \left(\Delta t,a\right)$ gives the probability of survival for Δt years, starting from age a (product of annual juvenile and adult survival probabilities). $B\left(a\right)$ is an individual's breeding state at age a, which here is individual i's age at $b_{j}$ ($a_{i}+b_{j}-y_{i})$, assuming she survives.

Then, using our definition of fecundity at age, Equation (2), we have

(5) $$\mathbb{P}\left[K_{\mathrm{ij}}=\mathrm{MOP}|a_{i},y_{i},b_{j},L_{i}=0,y_{i}<b_{j},\theta \right]=\frac{\Phi \left(b_{j}-y_{i},a_{i}\right)F\left(a_{i}+b_{j}-y_{i}\right)}{N_{b_{j},A}}.$$

If i is sampled after the birth of j ($b_{j}<y_{i}$), then i was either alive at j's birth or was not yet born, eliminating the need to account for survival or lethality terms. However, i may not have reached reproductive maturity by $b_{j}$. Letting $F\left(a⩽0\right)=0$,

(6) $$\mathbb{P}\left[K_{\mathrm{ij}}=\mathrm{MOP}|a_{i},y_{i},b_{j},b_{j}<y_{i},\theta \right]=\frac{F\left(a_{i}-\left(y_{i}-b_{j}\right)\right)}{N_{b_{j},A}}.$$

##### Maternal half‐sibling pairs (XmHSPs)

To find probabilities of cross‐cohort maternal half‐sibling pairs (XmHSPs), we check whether individual k and individual l have the same mother. We impose the following criteria:

1. $b_{l}>b_{k}$ (avoiding double counting);
2. $b_{k}\neq b_{l}$ (because walruses give birth to a single offspring at a time);
3. $b_{k}⩾2000$ and $b_{l}⩾2000$ (birth years must be greater than or equal to 2000, because population dynamics starts at 2000).

If we call m the mother of k, what is the probability that l's mother was m? We know that m was alive, mature, and in breeding state S2 at k's birth, and that m survived at least one more year after k's birth, otherwise k would not have survived (through its dependency on its mother) and would not have been sampled. In order for m to also be l's mother, three conditions must be met:

1. m survives until $b_{l}+1$, because we know l survived to be sampled at 1+ years of age;
2. m is in breeding state S2 in $b_{l}$;
3. amongst all the females that are alive and in breeding state S2 in year $b_{l}$, m is the mother.

Let $\Phi \left(\Delta t\right)$ be the adult probability of surviving another Δt years, and recall Ψ is the breeding cycle transition matrix. The three‐element probability vector of an animal being in each state (S1, S2, S3) at time t is $p^{\left[t\right]}$. Then $p^{\left[t+1\right]}=\Psi p^{\left[t\right]}$. Now define $p^{\left[0\right]}={\left(0,1,0\right)}^{⊤}$ which is the three‐element probability vector of m's breeding state at k's birth (certain state S2), let $B_{m}\left(y\right)$ be m's actual breeding state in any year y, and recall ${\bar{\beta }}_{2}$ is the proportion of adult females in breeding state S2. Then

(7) $$\begin{array}{cc} \mathbb{P}\left[K_{kl}=\text{XmHSP|}b_{k},b_{l},\theta \right] & \\ \;=\mathbb{P}\left[K_{lm}=\mathrm{MOP}|B_{m}\left(b_{k}\right)=S2,m\;\text{alive}\;\mathrm{at}\;b_{k}+1,b_{l},\theta \right] & \\ =\frac{\Phi \left(b_{l}-b_{k}\right){\left[\Psi^{b_{l}-b_{k}}p^{\left[0\right]}\right]}_{2}}{N_{b_{l},A}{\bar{\beta }}_{2}}, & \end{array}$$

where ${\left[\Psi^{b_{l}-b_{k}}p^{\left[0\right]}\right]}_{2}$ is the second element of the vector, that is, the probability that *m* (given she was alive) was again in breeding state S2 at *l*'s birth.

HSPs are one of several “second‐order” kin pairs that are practically indistinguishable genetically hence cannot be identified directly and unambiguously. Fortunately, HSPs are demographically by far the most common when the birth gap used for comparing samples is short. When the birth gap approaches twice the age of first birth, though, grandparent–grandchild pairs (GGPs) become more prevalent. To mitigate this issue, we restricted the range of birth gaps considered in the model to those where GGPs are rare (or indeed impossible in our simulated data; that is, below twice the age of first birth plus two years).

##### Self‐recaptures (SPs)

Our stage‐structured model simplifies population dynamics, but we have to make an additional assumption about sampling selectivity to include self‐recaptures. Here, we assume selectivity varies only by stage (adult/juvenile), not by age within stage. We only consider female samples for self‐recapture, since males are prone to permanent emigration (Beatty et al., 2022), so do not yield readily interpretable inferences.

To compute stage‐structured self‐recapture probabilities in a manner analogous to kin capture probabilities, we retain only the first and last capture of each individual. This is a reasonable approximation for walruses because the self‐recapture rate is relatively low. We condition on age of the first sample $\left(a_{1}\right)$ but *not* explicitly on age of the second sample; instead, we condition on the second sample's developmental stage at sampling ($d_{2}$). This is necessary because our model is stage‐ rather than age‐structured. If $d\left(a\right)$ is a function that maps age to developmental stage, with 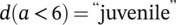 and 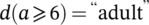, then we restrict our comparisons to pairs of samples collected in years $y_{1}$ and $y_{2}$ where

(8) $$d\left(a_{1}+\left(y_{2}-y_{1}\right)\right)=d_{2}.$$

If, based on age of the first sample $\left(a_{1}\right)$ and time elapsed between sampling events $\left(y_{2}-y_{1}\right)$ the first sample would have reached the developmental stage of the second sample ($d_{2}$; that is, the two could be the same animal), then we assume it is equally likely to be *any* of the females in that developmental stage in that year. Therefore, the probability that the first sample is the same individual as the second sample is the reciprocal of the developmental stage abundance. Additionally, we account for survival over the intervening years. The self‐recapture kinship probability between samples 1 and 2 (where $y_{1}<y_{2}$) is:

(9) $$\mathbb{P}\left[K_{12}=\mathrm{SP}|a_{1},y_{1},d_{2},y_{2},L_{1}=0,\theta \right]=\frac{I\left[d\left(a_{1}+\left(y_{2}-y_{1}\right)\right)=d_{2}\right]\Phi \left(y_{2}-y_{1},a_{1}\right)}{N_{y_{2},d_{2}}}.$$

The survival term $\Phi \left(y_{2}-y_{1},a_{1}\right)$ represents the probability of survival for Δt years as defined in *Kinship probabilities*. We also condition on the first sample being nonlethal (since the individual was subsequently recaptured). To obtain $N_{y_{2},d_{2}}$, we need either adult or juvenile abundance. Adult abundance is included in the population dynamics model, however, additional steps are required to deduce juvenile abundance. Assuming stable age composition, we show in Appendix S1: Section S3 that for walruses:

(10) $$N_{y,J}=N_{y,A}\frac{\lambda -\phi_{A}}{\lambda -\phi_{J}}\left({\left(\frac{\lambda }{\phi_{J}}\right)}^{5}-1\right),$$

where $\lambda =e^{r}$ is the relative annual population growth rate.

#### Pseudo‐likelihood

Given a real dataset, we would maximize the pseudo‐log‐likelihood that combines kinship probabilities and actual outcomes of all pairwise comparisons to estimate demographic parameters. To define the pseudo‐log‐likelihood, in brief, let $w_{\mathrm{ijk}}$ be “the data,” that is, the kinship outcome, for samples i and j and target kinship k: $w_{\mathrm{ijk}}=1$ if the actual kinship $K_{\mathrm{ij}}=k$, or $w_{\mathrm{ijk}}=0$ if $K_{\mathrm{ij}}\neq k$. As shown in Bravington et al. (2016), for “sparse sampling” CKMR where the population is large and the sampling fraction is correspondingly small, the comparisons are approximately statistically independent. Define $p_{\mathrm{ijk}}\left(\theta \right)=\mathbb{P}\left[K_{\mathrm{ij}}=k|z_{i},z_{j},\theta \right]$ to be the kinship probability for samples i and j, parameter values θ and covariates $z_{i}$ and $z_{j}$ (computed from, e.g., Equation (5)). In each case, the probability that $w_{\mathrm{ijk}}=1$ is on the order of the reciprocal of adult abundance, which is very small, and therefore the pseudo‐likelihood L is well approximated by a Poisson distribution with mean $p_{\mathrm{ijk}}\left(\theta \right)$:

(11) $$\begin{array}{c} w_{ijk}\sim \text{Poisson}\left(p_{ijk}\left(\theta \right)\right)\\ L\left(\theta ;w\right)=C\prod_{i<j;k\in K}e^{-p_{ijk}\left(\theta \right)}p_{ijk}{\left(\theta \right)}^{w_{ijk}}, \end{array}$$

where C is a constant and K are the kinship relationships being considered. Let $w=\left\{w_{\mathrm{ijk}};\forall i,j,k\right\}$, the possible combinations of samples and kin relationships; although in practice, some “impossible” comparisons are excluded (e.g., second‐order kin born a long time apart). Then, the pseudo‐log‐likelihood is:

(12) $$\log_{e}L\left(\theta ;\;w\right)=\Lambda \left(\theta ;\;w\right)=C+\sum_{i<j;k\in K}\left\{-p_{\mathrm{ijk}}\left(\theta \right)+w_{\mathrm{ijk}}\log_{e}\;p_{\mathrm{ijk}}\left(\theta \right)\right\}.$$

#### Design calculations

For design purposes, we use an analytical method to predict precision of the estimates expected under different sampling scenarios. The parameter uncertainty likely to result from proposed CKMR sampling designs can often be evaluated by calculation alone (Bravington et al., 2016, section 5). These calculations are adaptations of standard methods used to find the statistical information (i.e., derivatives) from the pseudo‐log‐likelihood, combined with enumerating the pairwise comparisons that would be available per covariate combination (which are limited here to: age or stage, sample year, and sex).

The statistical basis is given in Bravington et al. (2016), section 4. Following standard statistical practice, we approximate the parameter variance using the inverse of the (pseudo) Fisher Information $H\left(\theta_{0}\right)$ = $-E_{W}\left[d^{2}\Lambda \left(\theta_{0};W\right)/d\theta^{2}\right]$ (the negative expected Hessian over datasets evaluated at true parameter values $\theta_{0}$, which are taken from the simulation). As $\Lambda \left(W\right)$ is a sum of individual comparison terms, we can also write $H\left(\theta_{0}\right)=\sum_{i<j;k\in K}h_{\mathrm{ijk}}\left(\theta_{0}\right)$, where $h_{\mathrm{ijk}}\left(\theta_{0}\right)$ is the expected Fisher information *matrix* from a single comparison of type $\left(i,j,k\right)$. Further, Appendix S1: Section S4 shows that for Poisson random variables such as $w_{\mathrm{ijk}}$, we have

(13) $$h_{\mathrm{ijk}}\left(\theta_{0}\right)=4{\text{Δ}}_{\mathrm{ijk}}\left(\theta_{0}\right){\text{Δ}}_{\mathrm{ijk}}{\left(\theta_{0}\right)}^{⊤}\;\;\;\text{where}\;\;\;{\text{Δ}}_{\mathrm{ijk}}\left(\theta \right)=\frac{d\sqrt{p_{\mathrm{ijk}}\left(\theta \right)}}{d\theta }.$$

The vector ${\text{Δ}}_{\mathrm{ijk}}\left(\theta \right)$ can therefore be obtained for all $\left(i,j,k\right)$ by numerical differentiation of the probabilities calculated by the ICKMR model.

We now group across pairs with identical covariate values. Let $m\left(z\right)$ denote the number of samples with covariate combination z; the number of comparisons between two samples is $m\left(z_{1}\right)m\left(z_{2}\right)$ (ignoring double counting for the moment). The grouped version of the pseudo‐Fisher information can be written as

(14) $$H\left(m_{Z};\theta_{0}\right)=\sum_{z_{1},z_{2}\in Z;k\in K}\left(I\left(z_{1}<z_{2}\right)+\frac{1}{2}I\left(z_{1}=z_{2}\right)\right)m\left(z_{1}\right)m\left(z_{2}\right)h_{z_{1}z_{2}k}\left(\theta_{0}\right),$$

where the parentheses containing indicators handle double counting in the $m\left(z_{1}\right)m\left(z_{2}\right)$ product and $h_{z_{1}z_{2}k}\left(\theta_{0}\right)$ gives the Fisher information matrix for two samples with covariates $z_{1}$ and $z_{2}$ and kinship k. Z gives the collection of covariate combinations (analogous to K for the kinships) and $m_{Z}$ gives the sample sizes for those combinations (i.e., $m_{Z}$ is a vector as long as there are covariate combinations in Z and each element is the number of samples for that covariate combination).

We then invert matrix $H\left(m_{Z};\theta_{0}\right)$ to approximate the expected variance $V\left(m_{Z};\theta_{0}\right)$ of a parameter estimate. Uncertainty from any function of the parameters, $g\left(\theta \right)$, can then be approximated by the delta method:

(15) $$V\left[g\left(\theta \right);m_{Z},\theta_{0}\right]\approx \left[{\left.\frac{\mathrm{dg}\left(\theta \right)}{d\theta }\right|}_{\theta_{0}}\right]V\left(m_{Z},\theta_{0}\right){\left[{\left.\frac{\mathrm{dg}\left(\theta \right)}{d\theta }\right|}_{\theta_{0}}\right]}^{⊤}.$$

$\theta_{0}$ values for annual sample sizes and number of years of sampling come directly from our designs; however, the age–sex composition of the samples comes from our simulations.

The realized adult sample size (about 1100 per year for 2013–2017 and 2023, or 6600 total to date) is large enough relative to adult female abundance (~70,000; effectively more because of turnover during the years modeled) that ~5%–10% of samples are self/kin‐recaptures. This means that a considerable proportion of pairwise comparisons have predictable outcomes based on the results of other comparisons, breaking independence. The “sparse sampling” assumption of Bravington et al. (2016) is therefore not strictly justified, so the variance might be slightly over‐ or under‐estimated relative to our calculations. The direction is not entirely obvious, because finite population corrections will also affect the true variance, but we chose to eliminate redundant comparisons to err on the side of overestimating true variance. Specifically:

1. If an animal i was recaptured in multiple years, we only used i's last recapture in MOP and XmHSP comparisons.
2. If a sample j was identified as the offspring in a MOP, we did not use it in XmHSP comparisons (because the outcome of an XmHSP comparison between j and any other sample k could be deduced from j and k's MOP results; k and j are XmHSP if k was another offspring of j's mother).

Eliminating these comparisons means that we must adjust the effective sample sizes $m_{Z}$ accordingly. We used simulation results on the frequency of self‐recaptures and MOPs to determine how many samples would need to be eliminated and found that the effect is small for the scenarios we considered.

### Simulation for model checking and design scenarios

#### Simulations

To test our ICKMR model, we developed an individual‐based simulation with walrus life history, modified from the R (R Core Team, 2025) package fishSim (Baylis, 2019). The simulation is stochastic and operates on an annual basis. Individuals are tracked using unique identifiers allowing identification of kinship pairs in simulated samples. We ran the simulation from 1950 to 2030, using an initial population of 250,000 animals. These individuals are considered “founders” and do not have mothers or fathers. The age and sex structure of the initial population is determined by survival and fecundity rates used in the simulation (Table 1), which were based on estimated 2015 rates (Taylor et al., 2018). Parameters in Table 1 were adjusted to maintain the desired population growth rate ($e^{r}$). Individuals of breeding age mate randomly and males can potentially father more than one calf per year. Female reproduction is as described in *Life history and population dynamics*. Females that are in state S2 of the breeding cycle give birth to a single offspring with 1:1 sex ratio (Fay, 1982). There is no systematic age effect on female reproductive dynamics, except that they are guaranteed not pregnant at 4 years of age when they enter the breeding cycle (*Life history and population dynamics*), which slightly lowers effective fecundity for the first few years of adulthood until the Markov chain reaches equilibrium. We did not include senescence in our ICKMR model, but we did include it in our simulations so we could investigate effects of violating the assumption of “no senescence” in the ICKMR model.

**TABLE 1:** Demographic parameters for simulation under four scenarios (D0, D1, D2, and D3).

	Demographic scenario			
	D0	D1	D2	D3
Parameter	Null	Stationary	Decreasing	Increasing
Maximum age (AMAX)	37	37	37	37
Age at first birth for females (AFB)	6	6	6	6
Age of last birth for females (ALB)	37	29	29	29
Age of first fertility for males (AFF)	15	15	15	15
Young‐of‐the‐year (age 0 calf) survival	0.7	0.7	0.66	0.7
Juvenile survival (ages 1–5)	0.9	0.9	0.85	0.925
Reproductive adult female survival (ages 6 to ALR)	0.9622	0.99	0.985	0.99
Nonreproductive adult female survival (ages ALR to AMAX)	NA	0.55	0.5	0.55
Probability of breeding at 2‐year interval ($\psi_{2}$)	0.1	0.1	0.1	0.1
Probability of breeding at 3‐year + interval ($\psi_{3}$)	0.5	0.5	0.5	0.5
Resulting rate of increase (*r*)	0	0	−0.02	+0.01

*Note*: Scenario D0 was used to check the model code, whereas the other scenarios included reproductive senescence and were used to evaluate study design for a population that was either stationary (D1), decreasing (D2), or increasing (D3).

In sampling years, captures are simulated according to either historical or planned future sample sizes (Table 2). Females are available to be sampled at any age 1+, while males are available for sampling from ages 1–5 only, because adult males do not tend to travel to the Chukchi Sea in the summer. After sampling, some individuals die (according to age and/or sex‐specific mortality rates, Table 1). If a female with a YOTY dies, her calf also dies. Individuals automatically die if they reach the maximum age. Living individuals then have their age incremented.

**TABLE 2:** Details of sampling scenarios. For reference, scenarios are labeled S0–S8 with a description of effort.

Sampling scenario	Description	Effort per year					
		2023	2024	2025	2026	2027	2028
S0	Null: 100% effort 2023–2027	1	1	1	1	1	0
S1	Reality +100% effort 2025	1	0	1	0	0	0
S2	Reality +100% effort 2025–2026	1	0	1	1	0	0
S3	Reality +100% effort 2025–2027	1	0	1	1	1	0
S4	Reality +100% effort 2025–2028	1	0	1	1	1	1
S5	Reality +75% effort 2025	1	0	0.75	0	0	0
S6	Reality +75% effort 2025–2026	1	0	0.75	0.75	0	0
S7	Reality +75% effort through 2027	1	0	0.75	0.75	0.75	0
S8	Reality +75% effort through 2028	1	0	0.75	0.75	0.75	0.75

*Note*: Effort per year is indicated as either 0, 0.75 (75%), or 1 (100%) effort as described in *Simulation for model checking and design scenarios*. Each scenario was evaluated with and without the substitution of 500 lethal samples per year.

The breeding probability/birth rate is confounded with the YOTY survival rate. Because only samples from age 1 onwards are considered, only the product (nominal breeding probability rate × nominal YOTY survival) affects the simulated samples, not the two constituent parameters. The simulation then proceeds to the following year.

#### Model checking

To evaluate agreement between the simulation and ICKMR model, we simulated 50 replicate datasets with demographic parameters under a null scenario (D0, Table 1), and we simulated historical and future sampling according to realized or target sample sizes by age class, with effort per year constant at the 2023 level (S0, Table 2). The population dynamics model in the simulations is close (but not identical) to the ICKMR model because the simulation includes a definite maximum age, whereas the ICKMR model does not. We checked each of the simulated datasets against the ICKMR model for: observed (i.e., simulated) and expected numbers of kin pairs in different categories (MOPs, XmHSPs, and SPs); observed versus expected year gaps between half‐sibling pairs, unbiasedness of the log‐likelihood derivatives at the true parameter values, and parameter bias. These comparisons enabled us to evaluate whether the simulation and ICKMR models were consistent and whether simplifications made in the ICKMR model were acceptable. Refer to Appendix S1: Section S5 for details.

#### Scenarios

We evaluated the performance of ICKMR under different demographic and sampling scenarios. The demographic scenarios were a stationary population (D1), a slightly decreasing population (D2), and a slightly increasing population (D3) (Table 1). These values were chosen because they represent the credibility limits and point estimate for the 2015 walrus population growth rate based on an integrated population model (Taylor et al., 2018). We simulated historical sampling (2013–2017, when the first generation of research expeditions took place) according to realized sample sizes by age and sex (Beatty et al., 2022). We simulated possible reductions in future sampling effort, either by reducing the number of sampling years or by reducing the amount of sampling effort within years (S1–S8; Table 2). For simulated captures between 2023 and 2028, we estimated an expected overall sample size of 1600 per year with 100% effort (i.e., a 4‐week research expedition). We estimated that 75% effort (a 3‐week research expedition) would result in an expected sample size of 1200. Planned sampling went ahead in 2023 but not in 2024, so we modified simulated sampling scenarios 1–8 to represent the “reality” of 100% survey effort in 2023 and 0% survey effort in 2024.

The FWS Walrus Harvest Monitoring Program (WHMP) monitors the walrus harvest each year in two coastal communities in Alaska, which comprises 84% of total Alaska Native subsistence harvest (MacCracken et al., 2017). WHMP collects demographic data and biological samples from harvested animals. To assess the relative value of samples from harvested animals (versus biopsy samples from live individuals), we simulated each scenario without (L1) and with (L2) the substitution of 500 live biopsy samples with 500 lethal samples in sampling years 2023–2028.

With three demographic scenarios, eight sampling scenarios, and two lethality scenarios, this resulted in a total of 48 simulated datasets from which to evaluate survey design. Given the relatively large population size and large number of samples, we did not expect key properties of simulated datasets to differ substantially due to random variation. This was confirmed by model checking (refer to Appendix S1: Section S5). Therefore, we evaluated a single realization of each simulated scenario.

Beatty et al. (2022) achieved a CV of 0.19 on total abundance in a 5‐year study. With this result in mind, we compared the estimated CVs for adult female abundance derived from IMR and ICKMR models to precision benchmarks of CV = 0.20, CV = 0.10 (representing a 50% reduction in CV), and CV = 0.05 (representing a 75% reduction in CV). Thus, we evaluated the performance of our IMR and ICKMR models relative to the performance of a multievent model with sampling effort over 5 years (2013–2017).

## RESULTS

### Adult female abundance

**FIGURE 2:**
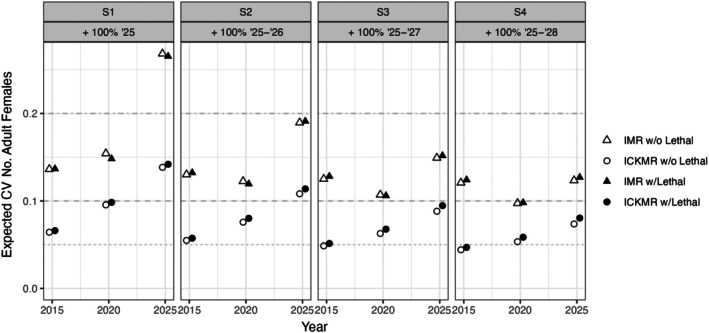
Expected CV of adult female abundance (vertical axis) in different years (horizontal axis) under different sampling scenarios (panel columns) for a simulated stationary population. For clarity, points have been jittered horizontally. Triangular points represent expected CVs from IMR alone, while circular points show expected CVs with ICKMR. The inclusion of lethal samples is indicated by filled (lethal samples substituted) or open (no lethal samples) points. The gray horizontal dot‐dashed, dashed, and dotted lines at CV = 0.2, 0.1, and 0.05 respectively represent decision‐making thresholds.

Across all sampling scenarios, ICKMR gave substantially more precise abundance estimates than IMR alone (Figure 2). This was also true across demographic scenarios (refer to Appendix S1: Figure S4 and Appendix S1: Table S4). The mean absolute decrease in CV on adult female abundance in paired scenarios with IMR and ICKMR was 7% for a stationary population, 4% for a decreasing population, and 8% for an increasing population. These represent relative decreases in CV of 47%, 45%, and 47%, respectively. Refer to Appendix S1: Table S4 for expected CVs of adult female abundance across all demographic and sampling scenarios with and without the substitution of lethal samples and use of CKMR.

The demographic scenarios (refer to Table 1) affected expected precision. With a declining population and smaller resulting population size during target inference years, there is less competition to be the kin of any given sample; therefore, the number of kin pairs is higher, reducing the expected CV for a given sample size. The opposite happens with an increasing population.

**FIGURE 3:**
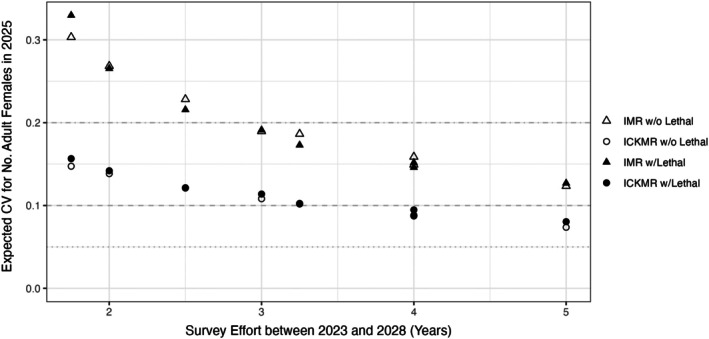
Total survey effort between 2023 and 2028 (in number of years, which may be a combination of calendar years of effort and fractional effort per year, horizontal axis) versus expected CV for adult female abundance in 2025 with IMR (triangular points) or with ICKMR (circular points) and with (filled) and without (open) the substitution of lethal samples for a simulated stationary population. The horizontal dot‐dashed, dashed, and dotted lines at CV = 0.2, 0.1, and 0.05 respectively represent decision‐making thresholds.

The simulated sampling scenarios resulted in between 1.75 and 5 years of total survey effort between 2023 and 2028 (where total survey effort is a combination of years of effort and effort per year, which may be fractional, and where 5 years of total survey effort between 2023 and 2027 was the original plan; Figure 3). In general, expected CVs on adult female abundance decreased with increasing number of total sampling years (Figure 3). For a simulated stationary population and with a target CV of 0.2 (similar to CV = 0.19 from the analysis in Beatty et al., 2022) on estimates of adult female abundance in 2025, sufficient precision was achieved in all sampling scenarios with ICKMR with or without the substitution of lethal samples (Figure 2 and Figure 3). For IMR with or without the substitution of lethal samples at least 3 years of total survey effort would be required to achieve a CV of 0.2. With a target CV of 0.1, ICKMR could achieve sufficient precision with 4 years of total survey effort, while IMR alone would not achieve this precision even with 5 years of total survey effort.

The simulated substitution of 500 lethal samples per sampling year slightly changed expected precision in abundance estimates (the mean change in CV with versus without lethal samples was <1% across all demographic and sampling scenarios, with a maximum increase of 3%). For ICKMR, expected CVs on adult female abundance were consistently higher when lethal samples were used, but the magnitude of the difference was small (mean increase of 0.14%). This suggests that lethal samples are almost equally valuable for walrus ICKMR, and the substitution of lethal samples for live biopsy samples could reduce required expedition length (500 samples ≈1/3 of samples expected during a 4‐week expedition).

### Demographic parameters

The simulated values of adult female survival and post‐senescent adult female survival (Table 1) resulted in effective survival from age 6–37 of 0.96, 0.95, and 0.96 for stationary, decreasing, and increasing populations, respectively. Depending on demographic scenario, sampling scenario, and substitution of lethal samples, the expected SEs on adult female survival ranged from 0 to 0.03. When estimated with ICKMR, the expected SEs on adult female survival were always lower than when IMR alone was applied (mean decrease in SE = 0.01). The simulated values of juvenile female survival from age 1–5 were 0.9, 0.85, and 0.925 (Table 1). Across demographic scenarios, sampling scenarios, and the substitution of lethal samples, the expected SEs on juvenile female survival ranged from 0.02 to 0.06. The mean expected decrease in SE on juvenile female survival with ICKMR was 0.01. Across all demographic and sampling scenarios, the simulated proportion of adult females in breeding state S2 (calving) was 0.26. The mean expected SEs on the proportion of adult females in breeding state S2 across demographic, sampling and lethality scenarios was 0.11 (range 0.01–0.31). The expected SEs on the proportion of adult females in breeding state S2 were notably lower when ICKMR was used compared with IMR (mean decrease in SE = 0.19). Refer to Appendix S1: Table S3 for expected SEs of life history parameters across all demographic and sampling scenarios with and without the substitution of lethal samples and use of ICKMR.

## DISCUSSION

We developed an ICKMR study design, with walrus as a case study, as an example for other researchers embarking on this evolving type of study. We investigated whether using ICKMR could increase expected precision in estimates of adult female walrus population size. To do this, we developed an ICKMR model with individual recaptures as a kin type (self‐pairs, SP) in addition to mother–offspring and cross‐cohort maternal half‐sibling pairs (MOPs and XmHSPs). We made simplifying assumptions for tractability. For example, we decided to exclude paternal kinships and not to model males at all because there would be minimal information in the data and extra complications in the modeling; we opted for a stage‐structured (rather than age‐structured) model, assuming unselective sampling by age within stage (which may not be particularly accurate for juveniles). In the future, a fully age‐structured version of the model would simplify the kinship probabilities for the self‐recapture data. We further assumed quasi‐equilibrium population dynamics across the period 2000–2028, with a constant rate of population change and stable age composition. This is a simplification during at least part of the time frame (Taylor et al., 2018). Nevertheless, given that our general purpose was to investigate sample size requirements, we believe our simplifications were reasonable.

The walrus project was initially planned with 5 years of total survey effort between 2013 and 2017 and another 5 years planned between 2023 and 2027. Because the 2013–2017 and 2023 surveys went ahead and the 2024 survey did not, we considered those years as fixed in our design scenarios. For all demographic scenarios, we found that expected relative CVs on adult female abundance were substantially (>30%) and consistently lower when using ICKMR than when using IMR. Our results indicated that by adding CKMR, a CV of <0.2 on estimates of adult female population size in 2025 could be achieved with 1.75 years of survey effort between 2023 and 2025, whereas IMR alone would require at least 3 years of total survey effort between 2023 and 2026 (with planned sample sizes per year of 1600; Figure 3). Because this expected CV applies to adult females only, decision makers may wish to set a lower target CV; for example, with a target CV of 0.1, 4 years of total survey effort would be required with ICKMR but would not be achievable within 5 years with IMR alone. Estimates of adult female and juvenile female survival, and of the proportion of adult females in breeding state S2 (calving), were also improved with the addition of CKMR.

Lethal samples can be incorporated into both IMR and CKMR analyses. In this study, we considered lethal samples as a potential replacement for some live samples, and assumed that lethal and nonlethal samples were similar in terms of ERO. Partial substitution of lethal samples for nonlethal (biopsy) samples resulted in similar precision on abundance estimates. In previous years, 50 samples per year were collected from harvested animals by the FWS WHMP. However, the total harvest in Alaska and Russia is estimated to number ≈4210 walruses per year (mean for 2016–2020; US Fish and Wildlife Service, 2023). Approximately 400 harvested samples would be needed to reduce each cruise by one week or 1600 samples would be needed to remove the need for an entire cruise. We did not investigate the impact of using exclusively harvested samples in place of one or more survey years, nor did we investigate the potential consequences of hunter preferences (e.g., if hunters preferentially target large adult females, and those females tend to be more fecund, the ERO of lethal and nonlethal samples may not be the same). In the longer term, using exclusively lethal samples could lead to lower precision in estimates of abundance, because lethal samples cannot go on to be self‐recaptures or future parents. Using samples from harvested walruses in combination with nonlethal samples collected from wild animals during research cruises can increase cost efficiency by reducing the need for extended at‐sea operations, thereby lowering logistical expenses associated with vessel charters and personnel time. Additionally, partial reliance on harvested samples mitigates disturbance to live walruses by decreasing the need for direct interactions with animals in the wild. This approach also strengthens collaboration with Alaska Native hunters and co‐management partners, fostering cooperative research efforts that align with subsistence practices and local ecological knowledge. For example, further work could be done in collaboration with the WHMP to better understand hunter preferences and to incorporate these into the CKMR model. Such partnerships are essential for long‐term monitoring and effective management of the species. Furthermore, the ongoing contribution of the WHMP to walrus abundance estimation provides a strong justification for maintaining the program, ensuring that robust population assessments continue to inform conservation and management decisions.

The results presented here all assume that age is accurately measured for each sample, using a DNA methylation‐based “epigenetic clock.” Although epigenetic age has been shown to work fairly well in a variety of species, including walruses as in Robeck et al. (2023), and further calibration studies are ongoing to improve cost and precision, epigenetic age is not perfectly precise. Failure to allow for any aging error in CKMR will certainly lead to bias; for example, the birth gap between XHSPs will be systematically overestimated, so that mortality rates will be underestimated. However, as long as the error variance of estimated age is known, it is possible to allow for aging error within the CKMR probability formulas, using weighted sums over kinship probabilities at different true ages. This should eliminate bias (Petersma et al., 2024), and Thomson et al. (2020) followed this approach for school shark *(Galeorhinus galeus)* using vertebral ages rather than epigenetic ages. In the case of ICKMR, the information from recaptured individuals will also be useful in resolving ages, because the interval between sampling will be known. Nevertheless, in severe cases, uncertainty about age can drastically limit the ability to gain information from CKMR, even when the number of kin pairs found is high and the model is adjusted properly, as noted from practical experience by Trenkel et al. (2022). We expect to include aging error in our ICKMR model when data are available and expect that doing so will reduce precision compared to having hypothetical perfect age information. The loss of precision can be investigated through our design framework, but we opted not to include it in our design calculations (i.e., we assumed that there is no error), because we do not yet know how large the errors will be. Design calculations can be easily re‐run when better estimates of aging error are available. For that purpose, a fully age‐structured, rather than stage‐structured model, would avoid the need to map uncertainty in age estimates to uncertain developmental stages.

The basic assumptions of CKMR are that each animal had one mother and one father, and that the types of close‐kin used in the model (generally first‐ and/or second‐order) can be reliably identified genetically. While most vertebrates meet these requirements, the practical considerations of sampling mean that it would not be sensible to apply CKMR to some species and populations. Design exercises like the one presented here can help quantify the cost and effort needed to achieve sufficient precision in quantities of interest using CKMR.

Because CKMR is a flexible modeling framework, and because sampling can be done in so many different ways, it is almost impossible to make absolute pronouncements about a species' suitability for CKMR, except in relation to some particular sampling scheme. For example, lethal sampling of persistent family groups, as in wolves (Canidae) or killer whales (*Orcinus orca*), would be unlikely to yield useful results. CKMR tends to work best for relatively large, well‐mixed populations, where sampling is sparse and approximate independence of comparisons is reasonable (refer to Bravington et al., 2016 for additional details). For any pairwise comparison used in the model, there should be no unmodeled correlation between sampling probability and reproduction, or between the event of an individual's being sampled and its expected number of sampled close‐kin. Sometimes this can be achieved by excluding certain pairwise comparisons from the model (e.g., between animals sampled close together in space and time, as for school shark in Thomson et al., 2020); sometimes by building a more elaborate model that conditions on covariates like place and time of sampling, thus avoiding the “unmodeled” issue. Here, for walruses, we assumed that if a mother is sampled, any accompanying offspring are likely nearby and therefore have increased sampling probability, which would violate the condition above. Therefore, we did not compare possible offspring and mothers sampled in the same year unless the potential offspring was definitely not accompanying its mother; that is, the offspring was sampled as an adult. These examples underscore the need to work closely with biologists to incorporate accurate information about the life history of the species of interest in CKMR model development.

Results described in this paper fully leverage CKMR to further advance population ecology. We demonstrate how self‐recaptures can be combined with CKMR in a unified model and substantially increase precision in estimates of population size and demographic parameters compared to IMR alone. We provide an example of ICKMR study design, including model development, model checking, and design calculations, and show how simulated data can be used to evaluate different proposed survey designs. While we used walrus as a motivating example, we expect that ICKMR can be used for estimating population parameters of interest across a range of taxa.

## AUTHOR CONTRIBUTIONS

Conceptualization: Eiren K. Jacobson, Mark V. Bravington, Rebecca L. Taylor, Irina S. Trukhanova, David L. Miller, William S. Beatty. Data curation: Eiren K. Jacobson. Formal analysis: Eiren K. Jacobson, Mark V. Bravington. Funding acquisition: Rebecca L. Taylor, Irina S. Trukhanova, William S. Beatty. Investigation: Eiren K. Jacobson, Mark V. Bravington. Methodology: Eiren K. Jacobson, Mark V. Bravington. Project administration: Eiren K. Jacobson, David L. Miller, William S. Beatty. Software: Eiren K. Jacobson, Mark V. Bravington, David L. Miller. Supervision: Eiren K. Jacobson, David L. Miller, William S. Beatty. Validation: Eiren K. Jacobson, Mark V. Bravington, Rebecca L. Taylor. Visualization: Eiren K. Jacobson, Mark V. Bravington. Writing – original draft: Eiren K. Jacobson, Mark V. Bravington, Rebecca L. Taylor. Writing – review and editing: Eiren K. Jacobson, Mark V. Bravington, Rebecca L. Taylor, Irina S. Trukhanova, David L. Miller, William S. Beatty.

## CONFLICT OF INTEREST STATEMENT

The authors declare no conflicts of interest.

## Supporting information


Appendix S1.

## Data Availability

Code (Jacobson, 2026) is available in Zenodo at https://doi.org/10.5281/zenodo.18782247.
