# Supplementary material for: Combining individual and close‐kin mark–recapture to design an effective wildlife population survey
Source: Ecology. 2026 Jun 22;107(6):e70377. doi: 10.1002/ecy.70377 (PMC13287309; doi:10.1002/ecy.70377)
Supplement: Supplementary file 1 — Appendix S1. [file ECY-107-e70377-s001.pdf]

Appendix S1

Combining individual and close-kin mark–recapture to design an elective wildlife population survey

Eiren K. Jacobson, Mark V. Bravington, Rebecca L. Taylor, Irina S. Trukhanova, David L. Miller, William S. Beatty

Ecology

Any use of trade, product or firm names is for descriptive purposes only and does not imply endorsement by the United States Government.

Section S1 Additional notes on males

Pacific walrus *(Odobenus rosmarus divergens)* mating is lek-based, and walrus are strongly sexually dimorphic with males much larger. It is plausible that some males dominate the reproductive output and may do so for several years in a row. We will not have sufficient numbers of adult male samples for individual recapture, nor for directly finding father-offspring pairs (FOPs). Without FOPs, there is no way to link any measurable characteristic of adult males to their reproductive output (whereas with fish, for example, there is often a clear empirical relationship between adult size and parent-offspring pair (POP) probability, so that a size-fecundity relationship can be fitted within the close-kin mark–recapture (CKMR) model; refer to Davies et al., 2020). Thus, we do not know how many adult males are under-breeding, nor whether it is a lifelong or transitory condition (e.g., if just a few males perpetually dominate mating, or if most males do eventually dominate the lek but only for a few years).

The only male adults that are visible through cross-cohort paternal half-sibling pairs (XpHSPs) are the ones that are breeding successfully, and in the absence of any independent information on which ones those are (e.g., size composition in fish, linked to empirical evidence of size-fecundity relationship) then there is no way of relating the number of successful breeders to overall male abundance. While cross-cohort half-sibling pairs (XHSPs) are often informative about mortality (as for female walrus), based on finding fewer half-siblings (HSPs) separated by longer gaps, the rate at which male walruses would be captured is actually the rate of leaving the pool of successful breeders, which depends on both mortality and the persistence of individual status.

The only remaining source of information about male adult abundance might come from juvenile male samples that later go on to father other samples, of which there will be few. Again, only some of the surviving male adults might be reproductively active, so there is confounding between abundance and variability in individual persistent breeding success.

The situation is different with females, which are biologically constrained to produce at most one offspring every two-plus years. There is little reason to expect big and persistent differences in individual reproductive output; any short-term differences due purely to age and maturity can be accommodated within the model because adult age is measured and can be tracked in the population dynamics.

While there might be some biological insights to be gained from inspection of male-parent close-kin pairs when the full samples are available, there is unlikely to be sufficient information to address overall population dynamics, especially abundance. Thus, omitting adult male dynamics at the design stage

and not considering XpHSPs and FOPs by when checking kin pairs is likely appropriate and simplifies analyses.

Section S2 Further HSP complications

An issue with all second-order kin (e.g., half-sibling pairs, HSP), is that pairwise-kinship statistics are not currently powerful enough to completely distinguish them from third-order kin such as great-grandparent-grandchild (GGP). To handle this issue without bias, we set a threshold for the statistic that should almost completely exclude false positives from third-order kin and estimate empirically the proportion of true second-order kin that will be lost below the threshold (i.e., the false-negative rate) based on the observed distribution of kin-pair statistics. Only kin pairs that are above the threshold will be treated as HSPs, but the probability formula can be multiplied by the complement of the false-negative probability to compensate. Refer to Bravington et al. (2016) or Hillary et al. (2018) for more details. The false-negative rate depends both on the species and the genotyping method (in particular, the number of loci) and cannot be predicted in advance. However, based on our experience, we have identified 15% as a safe upper limit.

Determining that a pair is a HSP does not differentiate between mHSPs (maternal; shared mother) and pHSPs (paternal; shared father). This can be determined by genotyping the mitochondrial DNA (mtDNA; always inherited from the mother only) of known HSPs. If the genotypes are different, the descent must be paternal; if the genotypes are the same, descent is probably maternal, but could arise by chance in a few pHSP cases. However, in our experience, except for very small populations (hundreds of adults), mtDNA diversity has always been high enough that shared-mtDNA HSPs were able to be treated as mHSPs. We assume as much here.

Section S3 Derivation of juvenile abundance

Following notation from the rest of the paper, let the number of adults in year y be $N_{\mathrm{A},t}$ where adulthood means being aged α or older. The number next year will be $\lambda N_{\mathrm{A},y+1}$ where $\lambda = e^r$ and r is the rate of increase as in Eq. (1). That will be made up of survivors from adults at t, plus survivors from the incoming cohort of oldest juveniles, aged $\alpha - 1$. Thus

$$N_{y+1,\mathrm{A}} = \lambda N_{y,\mathrm{A}} = \phi_\mathrm{A} N_{y,\mathrm{A}} + \phi_\mathrm{J} N_{y,\alpha-1}. \qquad \text{(Equation S1)}$$

Rearranging, we have

$$N_{y,\alpha-1} = \frac{\lambda - \phi_\mathrm{A}}{\phi_\mathrm{J}} N_{y,\mathrm{A}}. \qquad \text{(Equation S2)}$$

We now need to infer the numbers in the other juvenile age-classes (not just $\alpha - 1$). Starting with the penultimate juvenile age-class, we have:

$$\begin{aligned}
N_{y,\alpha-1} &= \phi_\mathrm{J} N_{y-1,\alpha-2} && \text{(survival)}\\
N_{y,\alpha-1} &= \lambda N_{y-1,\alpha-1} && \text{(population growth)}\\
\implies N_{y,\alpha-2} &= \frac{\lambda}{\phi_\mathrm{J}} N_{y,\alpha-1}. && \text{(Equation S3)}
\end{aligned}$$

Similar relationships apply to each preceding juvenile age class, down to age 1. The total number of juveniles in year y, $N_{y,\mathrm{J}}$, is given by a sum from age $x = \alpha - 1$ down to age 1:

$$\begin{aligned}
N_{y,\mathrm{J}} = \sum_{x=1}^{\alpha-1} N_{y,\alpha-x} &= \sum_{x=1}^{\alpha-1} N_{y,\alpha-1} \left(\frac{\lambda}{\phi_\mathrm{J}}\right)^{x-1}\\
&= N_{y,\alpha-1} \sum_{x'=0}^{\alpha-2} \left(\frac{\lambda}{\phi_\mathrm{J}}\right)^{x'}\\
&= N_{y,\alpha-1} \frac{1 - (\lambda/\phi_\mathrm{J})^{\alpha-1}}{1 - \lambda/\phi_\mathrm{J}}, && \text{(Equation S4)}
\end{aligned}$$

using the standard result for a geometric sequence: $\sum_{i=0}^{n} ar^i = a\frac{1-r^n}{1-r}$. Substituting for $N_{y,\alpha-1}$ from Eq. (Equation S2), we have

$$N_{y,\mathrm{J}} = N_{y,\mathrm{A}} \frac{\lambda - \phi_\mathrm{A}}{\phi_\mathrm{J}} \frac{1 - \left(\frac{\lambda}{\phi_\mathrm{J}}\right)^{\alpha-1}}{1 - \frac{\lambda}{\phi_\mathrm{J}}}$$

$$= N_{y,\mathrm{A}} \frac{\lambda - \phi_\mathrm{A}}{\lambda - \phi_\mathrm{J}} \left(\left(\frac{\lambda}{\phi_\mathrm{J}} \right)^{\alpha-1} - 1 \right). \quad \text{(Equation S5)}$$

Now, for the case of Pacific walrus *(Odobenus rosmarus divergens)*, we know that $\alpha = 6$, so:

$$N_{y,\mathrm{J}} = N_{y,\mathrm{A}} \frac{\lambda - \phi_\mathrm{A}}{\lambda - \phi_\mathrm{J}} \left(\left(\frac{\lambda}{\phi_\mathrm{J}} \right)^{5} - 1 \right). \quad \text{(Equation S6)}$$

Section S4 Expected information from a single Poisson observation

Suppose the random variable W follows a Poisson distribution with mean $m(\boldsymbol{\theta})$, where $\boldsymbol{\theta}$ is a vector of unknown parameters. Then, up to an additive constant, the log-likelihood $\Lambda(W)$ is

$$\Lambda(W) = -m(\boldsymbol{\theta}) + W \log m(\boldsymbol{\theta}) \qquad \text{(Equation S7)}$$

and the score function is

$$\frac{d\Lambda(W)}{d\boldsymbol{\theta}} = -\frac{dm(\boldsymbol{\theta})}{d\boldsymbol{\theta}} + \frac{W}{m(\boldsymbol{\theta})}\frac{dm(\boldsymbol{\theta})}{d\boldsymbol{\theta}} = \frac{dm(\boldsymbol{\theta})}{d\boldsymbol{\theta}}\left(\frac{W}{m(\boldsymbol{\theta})} - 1\right). \qquad \text{(Equation S8)}$$

The expected Fisher Information about the ith and j^{th} components of $\boldsymbol{\theta}$, defined as $\text{FI}_{ij} \triangleq \mathbb{E}_W\left[\frac{d\Lambda(W)}{d\boldsymbol{\theta}_i}\frac{d\Lambda(W)}{d\boldsymbol{\theta}_j}\right]$, is

$$\begin{aligned}
\text{FI}_{ij} &= \mathbb{E}_W\left[\frac{dm(\boldsymbol{\theta})}{d\boldsymbol{\theta}_i}\frac{dm(\boldsymbol{\theta})}{d\boldsymbol{\theta}_j}\left(\frac{W}{m(\boldsymbol{\theta})} - 1\right)^2\right] \\
&= \frac{dm(\boldsymbol{\theta})}{d\boldsymbol{\theta}_i}\frac{dm(\boldsymbol{\theta})}{d\boldsymbol{\theta}_j}\frac{1}{m(\boldsymbol{\theta})^2}\mathbb{E}\left[(W - m(\boldsymbol{\theta}))^2\right] \\
&= \frac{dm(\boldsymbol{\theta})}{d\boldsymbol{\theta}_i}\frac{dm(\boldsymbol{\theta})}{d\boldsymbol{\theta}_j}\frac{1}{m(\boldsymbol{\theta})^2}\mathbb{V}[W] \\
&= \left(\frac{1}{\sqrt{m(\boldsymbol{\theta})}}\frac{dm(\boldsymbol{\theta})}{d\boldsymbol{\theta}_i}\right)\left(\frac{1}{\sqrt{m(\boldsymbol{\theta})}}\frac{dm(\boldsymbol{\theta})}{d\boldsymbol{\theta}_j}\right).
\end{aligned} \qquad \text{(Equation S9)}$$

By the chain rule, we have

$$\frac{d\sqrt{m(\boldsymbol{\theta})}}{d\boldsymbol{\theta}} = \frac{1}{2\sqrt{m(\boldsymbol{\theta})}}\frac{dm(\boldsymbol{\theta})}{d\boldsymbol{\theta}} \qquad \text{(Equation S10)}$$

Substituting into (Equation S9), we arrive at the desired result for the entire Fisher Information matrix:

$$\text{FI} = 4\frac{d\sqrt{m(\boldsymbol{\theta})}}{d\boldsymbol{\theta}}\frac{d\sqrt{m(\boldsymbol{\theta})}}{d\boldsymbol{\theta}}^{\top}. \qquad \text{(Equation S11)}$$

Section S5 Model checking

Close-kin pairwise probability formulae are usually simple in hindsight, but can be challenging to develop correctly. One way to reduce the risk of mistakes is to generate simulated datasets and check that the close-kin mark–recapture (CKMR) code is giving the expected results when known parameter values are inserted. CKMR simulation code looks completely different from kinship-probability code, and the chance of "making the same mistake twice" is therefore much less than with many statistical simulations. Robustness is improved even further if two different people are involved, one to simulate and one to write kinship-probability code. Even though simulation is not strictly necessary for most CKMR design exercises, simulation may be worth the additional effort to help the whole process, and that is the approach we took for Pacific walrus *(Odobenus rosmarus divergens)*. We found and fixed several mistakes this way, both in the CKMR code and in the simulation code.

There are various options for CKMR model checking when simulated datasets are available, but the approach of actually fitting a CKMR analysis to each simulated dataset is extremely slow compared to analytical evaluation. We used four checks implemented in R (R Core Team, 2025) and aimed at detecting gross errors; power to detect subtle mistakes is lower, but in our experience, subtle mistakes are less likely than big mistakes. The first two checks are based on single realizations of simulated data and are suitable as diagnostics when fitting to real data; the last two checks require multiple simulated datasets.

1. Observed and expected totals of sampled kin pairs of each type. If these totals do not match reasonably well, there must be a major inconsistency between model and simulations. The definition of "reasonably well" can be guided by the inherent Poisson variability. If an expected total is 227, say, then we would not expect to see observed total much outside, say, the 95% confidence limits for a Poisson distribution with mean (and therefore variance) 227. This can be roughly approximated by $227 \pm 2\sqrt{227}$ or about [197, 257]. Clearly, the expected total needs to be fairly large for this to have much power, so increasing the simulated sample size can be helpful for checking purposes.

2. Breakdown of observed and expected kin-pair totals across some covariate of interest. If the totals from the previous step are not matching well, then the breakdown may help identify sources of problems. For example, the distribution of birth gaps between XmHSPs is driven in

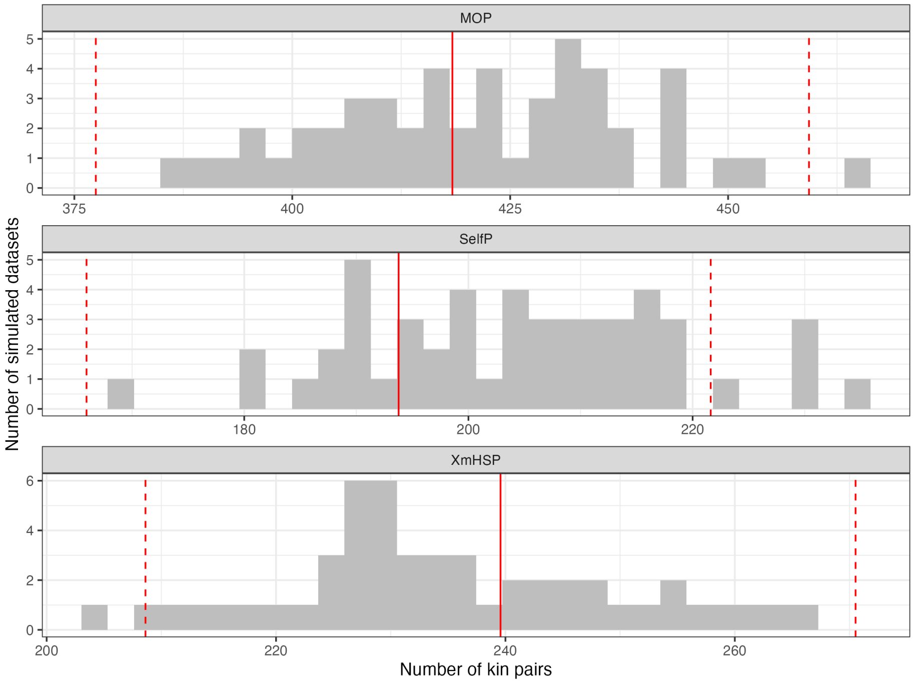

Figure S1: Number of kin pairs (horizontal axis) in 50 observed (simulated) datasets (vertical axis, grey bars) with the expected (modeled) number of kin pairs (red vertical line) and the expected number of kin pairs +/- 95% confidence intervals (red vertical dashed lines) for mother-offspring pairs (MOP), self-pairs (SP, denoted here as SelfP), and cross-cohort maternal half-sibling pairs (XmHSP). The observed numbers of kin pairs mostly fall within the 95% confidence intervals of the expected values.

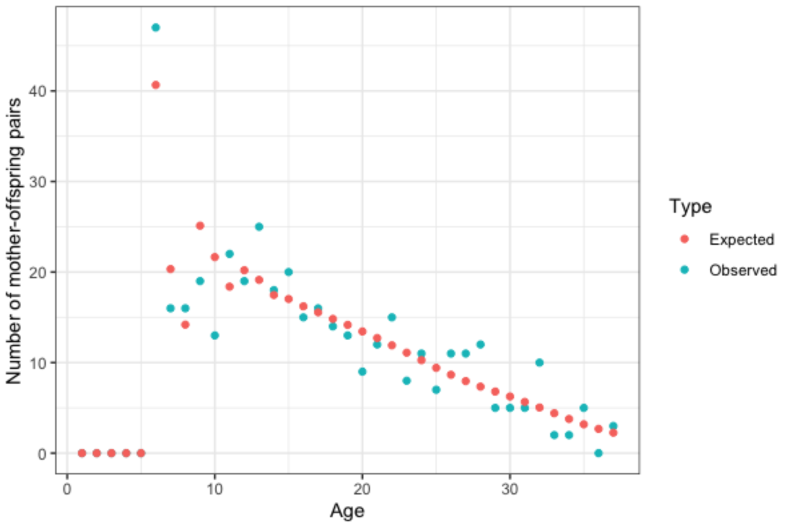

Figure S2: Expected (modeled, red points) versus observed (simulated, blue points) number of mother-offspring pairs (vertical axis) given mother's age at time of offspring's birth (horizontal axis). The observed number of pairs is not systematically higher or lower than expected, indicating that the treatment of the breeding cycle is consistent between the simulation and model.

the longer term by the adult rate mortality rate, so if observed and expected birth gaps do not correspond, then the treatment of mortality is likely inconsistent. Also, the number of mothers by age-at-birth should fluctuate over the first few years of adulthood because of the typically three-year breeding cycle (e.g., most 6yo have just given birth; most 7yo are still nursing last year's offspring), until the number of mothers by age-at-birth asymptotes because of the averaging effects of irregularities. If the observed and expected patterns do not match, then the breeding cycle treatment is inconsistent.

3. P-values of observed kin-totals by type, based on the Poisson distribution as above. Given a reasonable number of simulated datasets (say 20 or more), these should be roughly uniform across the interval [0,1]. It would require a large number of simulations to get a precise check here, but precision is not necessary because the goal is to pick up fairly coarse errors.

4. Looking at the mean and variance of the derivative of the pseudo-log-likelihood at the true parameter values $\boldsymbol{\theta}_0$ (something which can be calculated fairly quickly by numerical differentiation). The mean should be close to 0 and the variance determines what "close" might mean, given the number of simulations available. This checks the crucial "unbiased estimating equation" (UEE)

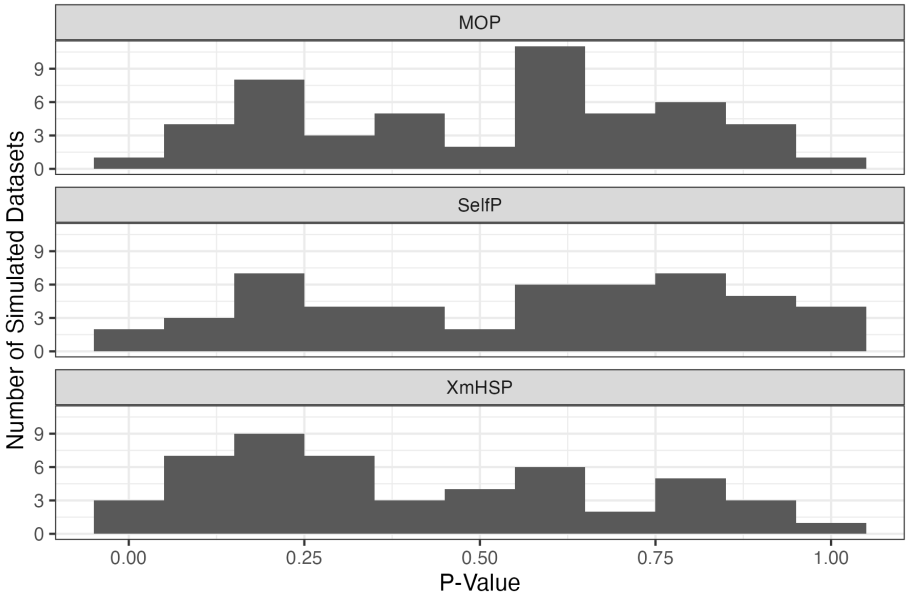

Figure S3: Histogram of p-values representing the probability of observing a number of kin pairs as or more extreme than the observed (simulated) value, given the expected (modeled) value generated given the sample size and age/sex composition, across 50 simulated datasets and three kinship types: mother-offspring pairs (MOPs), self-recaptures (SPs, denoted here as SelfP), and cross-cohort maternal half-sibling pairs (XmHSPs). and The p-values are approximately uniformly distributed, indicating that there is no systematic difference in production of kin pairs in the simulation versus the model.

Table S1: Mean and standard deviation of the derivative of the pseudo-log-likelihood at the true parameter values for model parameters across 50 simulated datasets. $\log_e N_{2015,A}$ is the log number of adult females in 2015, r is the rate of increase of the population, logitϕ_{A} is the logit of adult female survival, logit$(\phi_{\text{A}} - \phi_{\text{J}})$ is the logit difference between adult and juvenile female survival (because we estimated the difference parameter $\phi_{\Delta \text{J}} = \phi_{\text{A}} - \phi_J$ rather than separate adult and juvenile survival parameters), logitψ_1 is the logit probability of breeding on a two-year interval, logitψ_2 is the logit probability of breeding on a three-or-greater year interval.

	$\log_e N_{2015,A}$	r	logitϕ_{A}	logit$(\phi_{\text{A}} - \phi_{\text{J}})$	logitψ_1	logitψ_2
Mean	-9.28	-156	13.20	9.14	1.13	-0.13
Standard deviation	26.61	215.85	13.10	5.35	3.49	4.46

assumption required by most statistical estimation frameworks, including maximum-likelihood. If UEE does not hold, then by definition there is a mismatch between simulation and model. Results are shown in Table S1. Note that we can only produce results on the link scale (i.e., $\log_e$ or logit) because only in this case is the Hessian guaranteed to be positive definite.

The description so far implicitly assumes that the CKMR model (if working correctly) corresponds exactly to the data-generation mechanism in the simulations. However, the CKMR model may need to be simplified, especially for design purposes where the goal is just to make sure that sampling plans are sensible; developing a more complicated and realistic model can often be left until the real data appear. For example, we wanted to avoid reproductive senescence in the CKMR equations, so that all adults could be treated as a single block without requiring age-structured dynamics inside the model. Nevertheless, senescence likely occurs in walruses; therefore, simpler formulations should be checked to help avoid problems that prevent their utility. Simulated datasets can be used to estimate approximate bias in a slightly misspecified CKMR model, again without needing to do any estimation. The idea is to approximate the MLE for each dataset, based only on calculations using the true parameter value for the simulations. The MLE $\hat{\boldsymbol{\theta}}$ will by definition satisfy the equation $d\Lambda(W)(W)/d\boldsymbol{\theta}|_{\hat{\boldsymbol{\theta}}} = 0$ (where $\Lambda(W)(W) = \Lambda(W)(W)(\boldsymbol{\theta}; w)$, dropping arguments for compactness), and we can take a first-order Taylor expansion around the true value $\boldsymbol{\theta}_0$ to give

$$0 = \left.\frac{d\Lambda(W)(W)}{d\boldsymbol{\theta}}\right|_{\hat{\boldsymbol{\theta}}} \approx \left.\frac{d\Lambda(W)(W)}{d\boldsymbol{\theta}}\right|_{\boldsymbol{\theta}_0} + \left(\hat{\boldsymbol{\theta}} - \boldsymbol{\theta}_0\right) \left.\frac{d^2\Lambda(W)(W)}{d\boldsymbol{\theta}^2}\right|_{\boldsymbol{\theta}_0}$$

$$\implies \hat{\boldsymbol{\theta}} - \boldsymbol{\theta}_0 \approx - \left[\left.\frac{d\Lambda(W)(W)^2}{d\boldsymbol{\theta}^2}\right|_{\boldsymbol{\theta}_0} \right]^{-1} \left.\frac{d\Lambda(W)(W)}{d\boldsymbol{\theta}}\right|_{\boldsymbol{\theta}_0} \qquad \text{(Equation S12)}$$

The square-bracketed term can be replaced (to the same order of accuracy as the rest of the

Table S2: Mean absolute bias and bias relative to SD for model parameters across 50 simulated datasets. $\log_e N_{2015,A}$ is the log number of adult females in 2015, r is the rate of increase of the population, logitϕ_{A} is the logit of adult female survival, logit$(\phi_{\mathrm{A}} - \phi_{\mathrm{J}})$ is the logit difference between adult and juvenile female survival (since we estimated the parameter $\phi_{\Delta \mathrm{J}} = \phi_{\mathrm{A}} - \phi_J$ rather than separate adult and juvenile survival parameters), logitψ_1 is the logit probability of breeding on a two-year interval, logitψ_2 is the logit probability of breeding on a three-or-greater year interval.

	$\log_e N_{2015,\mathrm{A}}$	r	logitϕ_{A}	logit$(\phi_{\mathrm{A}} - \phi_{\mathrm{J}})$	logitψ_1	logitψ_2
Absolute bias	0.004	0.001	0.083	-0.585	-0.146	-0.045
Bias relative to SD	0.086	0.166	0.455	-1.73	-0.393	-0.154

approximation) by the *expected* Hessian, which is the crux of our design calculations and does not vary from one simulation to the next. Thus, the only quantity that has to be calculated per simulated dataset is $d\Lambda\left(W\right)\left(W\right)/d\boldsymbol{\theta}|_{\boldsymbol{\theta}_0}$, already required for the unbiased-estimating-equation check above. The estimated bias is the average across simulations of Eq. Equation S12. This is similar to the UEE check above, but with a change in focus: this time, we may be prepared to tolerate some small violation of UEE provided that it does not imply substantial bias on the parameter scale. In particular, if the estimated bias for the jth parameter (i.e. j^{th} component of $\boldsymbol{\theta}$) is below its sampling variability — say, if bias is less than 1 standard deviation, computed from the square-root of the diagonal of the inverse Hessian or $\sqrt{H^{-1}\left(j,j\right)}$— then there is little reason to worry about bias for that particular parameter. Table S2 show the results for our model. Again, note that we can only produce results on the link scale (i.e., $\log_e$ or logit) as only in this case is the Hessian guaranteed to be positive definite.

Based on the checks above, our estimation and simulation codes appeared consistent, and any bias induced by (among other minor things) ignoring senescence did not seem problematic. However, we only reached that position *after* going through the checking process several times to find and fix inconsistencies.

Section S6 Additional results

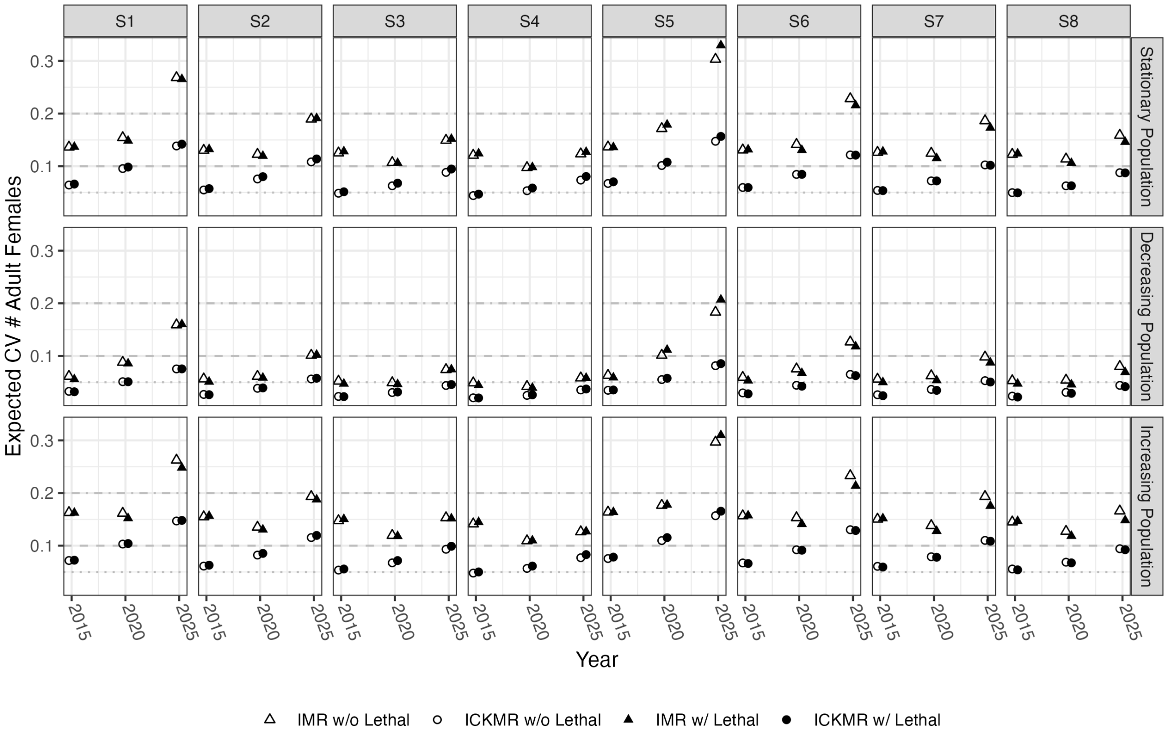

Figure S4: Expected CV of adult female abundance (vertical axis) in different years (horizontal axis) under different sampling scenarios (panel columns) for simulated stationary, decreasing, and increasing populations (panel rows). Demographic scenarios are defined in Table 1 and sampling scenarios in Table 2. For clarity, points have been jittered horizontally. Triangular points represent expected CVs from IMR alone, while circular points show expected CVs with ICKMR. The inclusion of lethal samples is indicated by filled (lethal samples substituted) or open (no lethal samples) points. The grey horizontal dot-dashed, dashed, and dotted lines at CV = 0.2, 0.1, and 0.05 respectively represent decision-making thresholds.

Table S3: Expected standard errors (SEs) on adult female survival, juvenile female survival, and the proportion of adult females in breeding state 2 under different demographic and sampling scenarios with and without the use of lethal samples and close-kin mark–recapture (CKMR). Demographic scenarios are denoted as D1 for a stationary population, D2 for a decreasing population, and D3 for an increasing population (refer to Table 1 for details). Sampling scenarios are defined in Table 2. $\hat{\phi_A}$ is estimated adult female survival, $\hat{\phi_J}$ is estimated juvenile female survival and $\hat{\psi_2}$ is the probability of being pregnant every 2 years.

Sampling Scenario	Demographic Scenario	Lethal Samples	CKMR	$\hat{\phi}_A$	$\hat{\phi}_J$	$\hat{\psi}_2$
S1	D1	No	No	0.02	0.06	0.28
		No	Yes	0.01	0.04	0.03
		Yes	No	0.02	0.05	0.29
		Yes	Yes	0.01	0.04	0.03
	D2	No	No	0.01	0.03	0.15
		No	Yes	0.01	0.02	0.01
		Yes	No	0.01	0.03	0.15
		Yes	Yes	0.01	0.02	0.01
	D3	No	No	0.02	0.06	0.29
		No	Yes	0.01	0.04	0.03
		Yes	No	0.02	0.05	0.29
		Yes	Yes	0.01	0.04	0.03
S2	D1	No	No	0.02	0.05	0.25
		No	Yes	0.01	0.03	0.03
		Yes	No	0.02	0.05	0.26
		Yes	Yes	0.01	0.04	0.03
	D2	No	No	0.01	0.03	0.13
		No	Yes	0.00	0.02	0.01
		Yes	No	0.01	0.03	0.13
		Yes	Yes	0.00	0.02	0.01
	D3	No	No	0.02	0.05	0.26
		No	Yes	0.01	0.03	0.03
		Yes	No	0.02	0.05	0.26
		Yes	Yes	0.01	0.03	0.03
S3	D1	No	No	0.01	0.04	0.22
		No	Yes	0.01	0.03	0.02
		Yes	No	0.01	0.04	0.23
		Yes	Yes	0.01	0.03	0.02
	D2	No	No	0.01	0.02	0.11
		No	Yes	0.00	0.02	0.01
		Yes	No	0.01	0.02	0.11
		Yes	Yes	0.00	0.02	0.01
	D3	No	No	0.02	0.04	0.23
		No	Yes	0.01	0.03	0.02
		Yes	No	0.02	0.04	0.23
		Yes	Yes	0.01	0.03	0.02

(Continued on next page...)

Sampling Scenario	Demographic Scenario	Lethal Samples	CKMR	$\hat{\phi}_A$	$\hat{\phi}_J$	$\hat{\psi}_2$
S4	D1	No	No	0.01	0.04	0.21
		No	Yes	0.01	0.03	0.02
		Yes	No	0.01	0.04	0.21
		Yes	Yes	0.01	0.03	0.02
	D2	No	No	0.01	0.02	0.10
		No	Yes	0.00	0.02	0.01
		Yes	No	0.01	0.02	0.10
		Yes	Yes	0.00	0.02	0.01
	D3	No	No	0.01	0.04	0.21
		No	Yes	0.01	0.03	0.02
		Yes	No	0.01	0.04	0.21
		Yes	Yes	0.01	0.03	0.02
S5	D1	No	No	0.02	0.06	0.29
		No	Yes	0.01	0.04	0.03
		Yes	No	0.02	0.06	0.30
		Yes	Yes	0.01	0.05	0.03
	D2	No	No	0.01	0.04	0.15
		No	Yes	0.01	0.03	0.02
		Yes	No	0.02	0.04	0.16
		Yes	Yes	0.01	0.03	0.02
	D3	No	No	0.02	0.07	0.30
		No	Yes	0.01	0.04	0.03
		Yes	No	0.02	0.06	0.31
		Yes	Yes	0.01	0.04	0.03
S6	D1	No	No	0.02	0.06	0.27
		No	Yes	0.01	0.04	0.03
		Yes	No	0.02	0.05	0.27
		Yes	Yes	0.01	0.04	0.03
	D2	No	No	0.01	0.03	0.14
		No	Yes	0.01	0.02	0.01
		Yes	No	0.01	0.03	0.13
		Yes	Yes	0.00	0.02	0.01
	D3	No	No	0.02	0.06	0.28
		No	Yes	0.01	0.04	0.03
		Yes	No	0.02	0.05	0.27
		Yes	Yes	0.01	0.04	0.03

(Continued on next page...)

Sampling Scenario	Demographic Scenario	Lethal Samples	CKMR	$\hat{\phi}_A$	$\hat{\phi}_J$	$\hat{\psi}_2$
S7	D1	No	No	0.02	0.05	0.25
		No	Yes	0.01	0.03	0.02
		Yes	No	0.02	0.05	0.25
		Yes	Yes	0.01	0.03	0.02
	D2	No	No	0.01	0.03	0.13
		No	Yes	0.00	0.02	0.01
		Yes	No	0.01	0.02	0.12
		Yes	Yes	0.00	0.02	0.01
	D3	No	No	0.02	0.05	0.26
		No	Yes	0.01	0.03	0.03
		Yes	No	0.02	0.05	0.25
		Yes	Yes	0.01	0.03	0.03
S8	D1	No	No	0.02	0.05	0.23
		No	Yes	0.01	0.03	0.02
		Yes	No	0.01	0.04	0.23
		Yes	Yes	0.01	0.03	0.02
	D2	No	No	0.01	0.03	0.12
		No	Yes	0.00	0.02	0.01
		Yes	No	0.01	0.02	0.11
		Yes	Yes	0.00	0.02	0.01
	D3	No	No	0.02	0.05	0.24
		No	Yes	0.01	0.03	0.02
		Yes	No	0.02	0.04	0.23
		Yes	Yes	0.01	0.03	0.02

Table S4: Expected coefficient of variation (CV) on adult female population size in 2015, 2020, and 2025 with different demographic and sampling scenarios and with and without the use of lethal samples and CKMR. Demographic scenarios are denoted as D1 for a stationary population, D2 for a decreasing population, and D3 for an increasing population (refer to Table 1 for details). Sampling scenarios are defined in Table 2. $\hat{N}_{y,\mathrm{A}}$ is the estimate of adult female abundance in year y.

Sampling Scenario	Demographic Scenario	Lethal Samples	CKMR	CV($\hat{N}_{2015,\mathrm{A}}$)	CV($\hat{N}_{2020,\mathrm{A}}$)	CV($\hat{N}_{2025,\mathrm{A}}$)
S1	D1	No	No	0.14	0.15	0.27
		No	Yes	0.06	0.10	0.14
		Yes	No	0.14	0.15	0.27
		Yes	Yes	0.07	0.10	0.14
	D2	No	No	0.06	0.09	0.16
		No	Yes	0.03	0.05	0.08
		Yes	No	0.06	0.09	0.16
		Yes	Yes	0.03	0.05	0.08
	D3	No	No	0.16	0.16	0.26
		No	Yes	0.07	0.10	0.15
		Yes	No	0.16	0.15	0.25
		Yes	Yes	0.07	0.10	0.15
S2	D1	No	No	0.13	0.12	0.19
		No	Yes	0.05	0.08	0.11
		Yes	No	0.13	0.12	0.19
		Yes	Yes	0.06	0.08	0.11
	D2	No	No	0.06	0.06	0.10
		No	Yes	0.03	0.04	0.06
		Yes	No	0.05	0.06	0.10
		Yes	Yes	0.03	0.04	0.06
	D3	No	No	0.15	0.14	0.19
		No	Yes	0.06	0.08	0.12
		Yes	No	0.16	0.13	0.19
		Yes	Yes	0.06	0.09	0.12
S3	D1	No	No	0.13	0.11	0.15
		No	Yes	0.05	0.06	0.09
		Yes	No	0.13	0.11	0.15
		Yes	Yes	0.05	0.07	0.09
	D2	No	No	0.05	0.05	0.07
		No	Yes	0.02	0.03	0.04
		Yes	No	0.05	0.05	0.07
		Yes	Yes	0.02	0.03	0.05
	D3	No	No	0.15	0.12	0.15
		No	Yes	0.05	0.07	0.09
		Yes	No	0.15	0.12	0.15
		Yes	Yes	0.06	0.07	0.10

(Continued on next page...)

Sampling Scenario	Demographic Scenario	Lethal Samples	CKMR	CV($\hat{N}_{2015,\mathrm{A}}$)	CV($\hat{N}_{2020,\mathrm{A}}$)	CV($\hat{N}_{2025,\mathrm{A}}$)
S4	D1	No	No	0.12	0.10	0.12
		No	Yes	0.04	0.05	0.07
		Yes	No	0.12	0.10	0.13
		Yes	Yes	0.05	0.06	0.08
	D2	No	No	0.05	0.04	0.06
		No	Yes	0.02	0.03	0.04
		Yes	No	0.04	0.04	0.06
		Yes	Yes	0.02	0.03	0.04
	D3	No	No	0.14	0.11	0.13
		No	Yes	0.05	0.06	0.08
		Yes	No	0.14	0.11	0.13
		Yes	Yes	0.05	0.06	0.08
S5	D1	No	No	0.14	0.17	0.30
		No	Yes	0.07	0.10	0.15
		Yes	No	0.14	0.18	0.33
		Yes	Yes	0.07	0.11	0.16
	D2	No	No	0.06	0.10	0.18
		No	Yes	0.03	0.06	0.08
		Yes	No	0.06	0.11	0.21
		Yes	Yes	0.04	0.06	0.09
	D3	No	No	0.16	0.18	0.30
		No	Yes	0.08	0.11	0.16
		Yes	No	0.16	0.18	0.31
		Yes	Yes	0.08	0.12	0.17
S6	D1	No	No	0.13	0.14	0.23
		No	Yes	0.06	0.08	0.12
		Yes	No	0.13	0.13	0.22
		Yes	Yes	0.06	0.08	0.12
	D2	No	No	0.06	0.08	0.13
		No	Yes	0.03	0.04	0.06
		Yes	No	0.05	0.07	0.12
		Yes	Yes	0.03	0.04	0.06
	D3	No	No	0.16	0.15	0.23
		No	Yes	0.07	0.09	0.13
		Yes	No	0.16	0.14	0.21
		Yes	Yes	0.07	0.09	0.13

(Continued on next page...)

Sampling Scenario	Demographic Scenario	Lethal Samples	CKMR	CV($\hat{N}_{2015,\mathrm{A}}$)	CV($\hat{N}_{2020,\mathrm{A}}$)	CV($\hat{N}_{2025,\mathrm{A}}$)
S7	D1	No	No	0.13	0.12	0.19
		No	Yes	0.05	0.07	0.10
		Yes	No	0.13	0.12	0.17
		Yes	Yes	0.05	0.07	0.10
	D2	No	No	0.06	0.06	0.10
		No	Yes	0.03	0.04	0.05
		Yes	No	0.05	0.05	0.09
		Yes	Yes	0.02	0.03	0.05
	D3	No	No	0.15	0.14	0.19
		No	Yes	0.06	0.08	0.11
		Yes	No	0.15	0.13	0.18
		Yes	Yes	0.06	0.08	0.11
S8	D1	No	No	0.12	0.11	0.16
		No	Yes	0.05	0.06	0.09
		Yes	No	0.12	0.11	0.15
		Yes	Yes	0.05	0.06	0.09
	D2	No	No	0.05	0.05	0.08
		No	Yes	0.02	0.03	0.04
		Yes	No	0.05	0.05	0.07
		Yes	Yes	0.02	0.03	0.04
	D3	No	No	0.15	0.13	0.17
		No	Yes	0.06	0.07	0.09
		Yes	No	0.15	0.12	0.15
		Yes	Yes	0.05	0.07	0.09

References

Bravington, M. V., H. J. Skaug, and E. C. Anderson (2016). "Close-Kin Mark-Recapture". In: *Statistical Science* 31.2, pp. 259–274.

Davies, C. et al. (2020). *Next-generation Close-kin Mark Recapture: Using SNPs to identify half-sibling pairs in Southern Bluefin Tuna and estimate abundance, mortality and selectivity.* FRDC report 2016-044. CSIRO.

Hillary, R. et al. (2018). "Genetic relatedness reveals total population size of white sharks in eastern Australia and New Zealand". In: *Nature Scientific Reports* 8 (1), p. 2661.

R Core Team (2025). *R: A Language and Environment for Statistical Computing.* Version 4.5.2. Vienna, Austria: R Foundation for Statistical Computing.
